# Comparing the carbon footprint of fMRI data processing and analysis approaches

**DOI:** 10.1162/IMAG.a.36

**Published:** 2025-06-16

**Authors:** Nicholas E. Souter, Chris Racey, Nikhil Bhagwat, Reese Wilkinson, Niall W. Duncan, Gabrielle Samuel, Loïc Lannelongue, Raghavendra Selvan, Charlotte L. Rae

**Affiliations:** School of Psychology, University of Sussex, Brighton, United Kingdom; Sussex Neuroscience, University of Sussex, Brighton, United Kingdom; McConnell Brain Imaging Centre, The Neuro (Montreal Neurological Institute - Hospital), McGill University, Montreal, Quebec, Canada; Department of Physics and Astronomy, University of Sussex, Brighton, United Kingdom; Graduate Institute of Mind, Brain and Consciousness, Taipei Medical University, Taipei, Taiwan; Department of Global Health and Social Medicine, King’s College London, London, United Kingdom; Cambridge Baker Systems Genomics Initiative, Department of Public Health and Primary Care, University of Cambridge, Cambridge, United Kingdom; British Heart Foundation Cardiovascular Epidemiology Unit, Department of Public Health and Primary Care, University of Cambridge, Cambridge, United Kingdom; Victor Phillip Dahdaleh Heart and Lung Research Institute, University of Cambridge, Cambridge, United Kingdom; Health Data Research UK Cambridge, Wellcome Genome Campus and University of Cambridge, Cambridge, United Kingdom; Department of Computer Science, University of Copenhagen, Copenhagen, Denmark

**Keywords:** neuroimaging, fMRI, preprocessing, environmental sustainability, carbon footprint, climate change

## Abstract

We compared the carbon emissions of preprocessing and statistical analysis of fMRI data in software packages FSL, SPM, and fMRIPrep using an existing open access dataset. Carbon emissions for fMRIPrep were 30x larger than those of FSL, and 23x those of SPM. We also compared the scientific performance of each package, reflected by sensitivity to statistical activation. Overall, fMRIPrep demonstrated slightly superior statistical sensitivity to both FSL and SPM, with FSL also outperforming SPM. However, this pattern varied by brain region. Researchers analysing fMRI data can use these findings to inform their choice of software package, considering the carbon footprint of data processing alongside usability and quality of derived output. Researchers should be conscious of how and when tools that elicit heavy compute are used, minimising energy usage and subsequent file size when possible. Researchers developing and using such tools should consider the extent to which computationally expensive steps are necessary to produce high-quality results.

## 1 Introduction

Human activity is having radical, significant, and harmful impacts on the climate, including loss of biodiversity and increased risk of extreme weather events and natural disasters (IPCC, 2023). Due in part to human*inactivity*in addressing this crisis, evidence suggests that in 2024, global surface temperature exceeded 1.5^o^C above pre-industrial levels (Bevacqua et al., 2025;Cannon, 2025). While research has a crucial role to play in combatting the climate crisis, scientists must acknowledge the carbon footprint of research practices. One aspect of this footprint arises from computing. Both the processing and storage of data require energy, given the reliance on electricity to operate servers (Khalaj et al., 2016). Neuroscience research frequently relies on analysis of large datasets. For example, human neuroimaging research, using brain scanning techniques such as magnetic resonance imaging (MRI), includes image preprocessing in order to reshape data to standard templates, remove noise, and correct distortion; and statistical analysis of these preprocessed data. High-performance computing is useful for performing these tasks at scale in a time-efficient manner, and when demands on memory and other resources are high. The extent of compute power needed in human neuroimaging is likely to continue to grow with increased adoption of machine learning approaches (Anthony et al., 2020;Singh et al., 2022).

Although estimates vary, the information and communications technology (ICT) sector accounts for 1.4–3.9% of global emissions (Freitag et al., 2021;Malmodin et al., 2024). There are several strategies for reducing the carbon footprint of computing, combinations of which must be simultaneously adopted to achieve sustainability targets for the sector. First, decarbonisation of energy (increasing use of renewable sources). Second, development of energy-efficient hardware. Third, coding of computationally efficient software. And finally, behavioural change in software usage. For the latter two approaches, it is crucial to be able to estimate the carbon footprint of research computing (Lannelongue & Inouye, 2023;Lannelongue et al., 2023). The extent of this footprint has been measured for computationally demanding disciplines including astrophysics (Portegies Zwart, 2020), Earth observation (Anderson et al., 2024;Wilkinson et al., 2024), and bioinformatics (Grealey et al., 2022).

We previously estimated the carbon footprint of functional magnetic resonance imaging (fMRI) preprocessing in the commonly used software package fMRIPrep (Souter et al., 2024). fMRIPrep combines tools from a number of software packages (including FSL, which is also assessed here). Preprocessing 6 minutes of fMRI data used 0.12 kWh of energy, amounting to 0.03 kg of carbon dioxide equivalent (when run in the UK), and generated 5.6 GB of data for each participant. Across all 257 participants, this amounted to 7.5 kgCO_2_e, equivalent to driving approximately 43.1 miles (69.4 km) in a passenger car^1^. The hard drive storage of these data across participants amounted to 1.4 TB, with a carbon footprint of 14 kgCO_2_e per year (assuming 10 kg/TB/year^2^). Fortunately, switching off the default step of FreeSurfer surface reconstruction reduced emissions by 48% with no trade-off in preprocessing performance, assuming users do not need surface reconstruction output for subsequent analysis. Furthermore, up to 96% of files generated by fMRIPrep will not be used by many users in subsequent analysis, and can typically be safely deleted (Souter et al., 2023). Both steps in conjunction would reduce the footprint of computing to 3.9 kgCO_2_e, and of storage to 0.6 kgCO_2_e per year (a total saving of 79%). This demonstrates that simple actions can substantially reduce the energy needed for processing and storing data.

These results reflect one fMRI package, and are restricted to preprocessing. However, researchers in human neuroimaging rely on a wider suite of tools, and undertake a number of analytical steps. In the current study, we measured and compared the carbon footprint of (1) fMRI preprocessing and (2) statistical analysis using general linear models in the three packages of FSL (FMRIB Software Library;Woolrich et al., 2001), SPM (Statistical Parametric Mapping;Penny et al., 2006), and fMRIPrep (Esteban et al., 2019). This enabled us to benchmark the carbon footprint of computing in neuroimaging research and understand effects of software package choice. We also assessed how differences in carbon emissions across packages balance against output quality. This was assessed quantitatively using statistical activation in regions of interest, and qualitatively by comparing clusters implicated in group-level analysis of task activation.

It was predicted that emissions from preprocessing would be significantly larger than from first-level statistical analysis, across packages. The footprint of preprocessing data in fMRIPrep is also likely to be considerably larger than in FSL or SPM, given that fMRIPrep employs a number of best-practice but computationally expensive tools (Esteban et al., 2019). This increased computing power may coincide with superior data quality. Overall, this study aims to inform best practices for optimal neuroimaging pipelines that balance carbon emissions and performance.

The results reported in this paper have been reproduced by an independent statistician from the University of Sussex (https://osf.io/npv2d).

## 2 Methods

### 2.1 Preregistration

This project was preregistered on the OSF (https://osf.io/sqnbw) on January 19^th^, 2024. This preregistration was created using the Psychological Research Preregistration-Quantitative (PRP-QUANT) Template, version 2 (available athttps://www.psycharchives.org). Several deviations were made from the preregistration, detailed in the Supplementary Section “Deviations from preregistration.”

### 2.2 Participants

We used data from the publicly available UCLA Consortium for Neuropsychiatric Phenomics LA5c Study (Bilder et al., 2020;Gorgolewski et al., 2017;https://openneuro.org/datasets/ds000030/versions/1.0.0). A “data descriptor” of this project is provided byPoldrack et al. (2016). This dataset includes 272 participants including those with a diagnosis of ADHD (N = 43), bipolar disorder (N = 29), or schizophrenia (N = 50) as well as neurologically healthy controls (N = 130). Participants completed a series of MRI scans including structural scans, diffusion-weighted imaging, resting-state fMRI, and task-based fMRI for six tasks. We analysed functional data for the stop signal task only. Data for this same sample and task were used in prior comparisons of fMRIPrep and FSL (Esteban et al., 2019). Participants were excluded if they did not have data for a T1 structural scan or the single run of the stop signal task (N = 13). We also excluded participants who had no valid trials classified as successful go (N = 1) or successful stop (N = 1). Participants were excluded if their raw fMRI data had an abnormal native resolution (N = 9) compared with that of the rest of the sample (3 x 3 x 4mm). Inclusion of these participants would have complicated group-level comparison of data preprocessed in fMRIPrep. The final sample included 248 participants (107 female) with a mean age of 32.9 years (SD = 9.2).

### 2.3 Design

This study had a repeated measures design, with raw fMRI data for all 248 participants being run separately through FSL, SPM, and fMRIPrep.

### 2.4 Procedure

#### 2.4.1 Stop signal task

The procedure used for the stop signal task is described fully inPoldrack et al. (2016). Participants completed one run of this task during the fMRI scan. Each participant completed 128 trials; 96 “go” and 32 “stop.” Each trial was preceded by a 500 ms fixation cross. Participants were then presented with an arrow pointing left or right, indicating the response they should provide, which remained visible for 1,000 ms (the decision period). Using a button box in their right hand, participants were instructed to indicate the direction of the stimulus arrow on “go” trials. Participants were instructed to inhibit these button presses during “stop” trials, on which they heard a 500 Hz audio tone. The delay period between the arrow appearing and the auditory “stop” stimulus for a given trial increased by 50 ms intervals after previous successful inhibition of a motor response and decreased by 50 ms intervals following failed inhibitions. Audio stop signals lasted for 250 ms. Trials were separated by jittered null periods during which a blank screen was presented. Null periods ranged from 500 ms to 4,000 ms, with an average of 1,000 ms.

#### 2.4.2 fMRI data preprocessing

fMRI data for the same 248 participants were preprocessed separately in FSL fMRI Expert Analysis Tool (FEAT), SPM, and fMRIPrep. The specific steps implemented in each package are documented inTable 1and are summarised briefly below.

**Table 1.** Preprocessing and statistical analysis parameters used for data processed in FSL, SPM, and fMRIPrep.

Stage	Processing step	FSL	SPM	fMRIPrep
Preprocessing	Script	FEAT First-level analysis: Preprocessing	Batch (multiple modules)	Custom fMRIPrep command line
	Slice timing	None	None	None (—ignore slicetiming)
	Realignment/motion correction	Pre-stats: Motion correction: MCFLIRT	Realign: Estimate & Reslice: default settings	MCFLIRT (FSL)
	Segmentation	None	Segment: Save Bias Corrected to “Save Bias Corrected.” Deformation fields set to “forward,” otherwise default	FAST (FSL) (—fs-no-reconall)
	Brain extraction (anatomical)	BET (prior to FEAT)	None	antsBrainExtraction.sh (ANTs)
	Brain extraction (functional)	Pre-stats: BET brain extraction	None	antsBrainExtraction.sh (ANTs)
	Intrasubject Coregistration	Registration: Linear, Normal search, BBR	Coregister: Estimate: Reference image = mean functional data, source image = T1w scan. Default settings	FLIRT (FSL), Linear
	Intersubject registration	Registration: Linear, Normal search, 12 DOF	Normalise: Write: using deformation field generated during “Segment,” otherwise default settings	antsRegistration (ANTs), Non-linear
	Analysis voxel size	3 x 3 x 4mm (determined from input functional image)	2 x 2 x 2mm ([2 2 2] set in Voxel size in Normalise: Write)	3 x 3 x 4mm (determined from input functional image)
	Smoothing	Pre-stats: Spatial smoothing FWHM (mm): 5	Smooth: FWHM 5 mm [5 5 5], otherwise default	AFNI 3DBlurInMask, FWHM 5 mm (performed outside fMRIPrep)
	Temporal filtering	Pre-stats: Temporal filtering: High-pass, cutoff = 100 seconds	fMRI model specification: Data & Design: High-pass filter = 128 seconds	FSL FEAT Pre-stats: Temporal filtering: High-pass, cutoff = 100 seconds
	Surface reconstruction	None	None	None (—fs-no-reconall)
	Distortion correction	None	None	None
First-level statistical analysis	Script	FEAT First-level analysis: Statistics	Batch (multiple modules)	FEAT First-level analysis: Statistics
	Model specification	Stats: Full model setup: EVs	fMRI model specification: Units for design = Seconds, Interscan interval = 2	Stats: Full model setup: EVs
	Motion parameters	Stats: Standard motion parameters	fMRI model specification: Multiple regressors: Realignment parameters file	Stats: Add additional confound EVs (trans_x, trans_y, trans_z, rot_x, rot_y, rot_z)
	Model estimation	Nothing to specify	Model estimation: Default settings	Nothing to specify
	Contrasts	Stats: Full model setup: Contrasts & F-tests	Contrast manager	Stats: Full model setup: Contrasts & F-tests
Second-level statistical analysis	Script	FEAT Higher-level analysis: Statistics	Batch (multiple modules)	FEAT Higher-level analysis: Statistics
	Model specification	Stats: Full model setup: EVs	Factorial design specification: One-sample t-test	Stats: Full model setup: EVs
	Model estimation	Nothing to specify	Model estimation: Default settings	Nothing to specify
	Contrasts	Stats: Full model setup: Contrasts & F-Tests	Contrast manager	Stats: Full model setup: Contrasts & F-Tests
	Second-level inference	Post-stats: Thresholding: Cluster: Z = 3.1, *p* = 0.05	Results Report: Thresholding: Cluster: *p* = 0.001, *p* = .05	Post-stats: Thresholding: Cluster: Z = 3.1, *p* = 0.05

Note: The parameter overview used here was inspired byTable 1fromBowring et al. (2019), who provide a quantitative comparison of MRI packages. Template scripts and files used for preprocessing and statistical analysis of data in each package are available on GitHub (https://github.com/NickESouter/fMRI-Computing-Footprint).

For FSL (version 6.0.4.), data were first run through the Brain Extraction Tool (BET). All other preprocessing steps were run in FSL FEAT, with settings kept at default. At this stage, we implemented “First-level analysis” for “Preprocessing” only (rather than “Full analysis”) in order to isolate the respective carbon footprints of individual-level preprocessing and statistical analysis.

For SPM (version SPM12), data were preprocessed in a batch using the modules listed inTable 1. Settings were largely kept at default as defined in the GUI, with the following exceptions: (1) during Segment, “Save Bias Corrected” was switched on and Deformation fields were set to “Forward,” (2) during Normalise: Write, deformation field generated during Segment was used, (3) during Smooth, FWHM was set to [5 5 5] rather than [8 8 8].

Preprocessing in fMRIPrep (version 22.1.1.) was run in a singularity container. The settings used in the fMRIPrep command line are summarised inSupplementary Table 1. Settings were largely kept at default, with the most notable exception being that FreeSurfer surface reconstruction was disabled. This was done to allow for a fairer comparison, given that surface reconstruction is not performed in FSL FEAT or SPM. Note that as shown in prior work (Souter et al., 2024), disabling surface reconstruction in fMRIPrep drastically reduces estimated carbon emissions, by an average of 48%. Further details on the steps implemented can be seen in the Supplementary Section “fMRIPrep citation boilerplate.” Given that smoothing is not included within fMRIPrep, we followed the procedure for smoothing used byEsteban et al. (2019). The preprocessed data for the stop signal task were smoothed using the AFNI (Analysis of Functional NeuroImages) tool 3dBlurInMask, with an FWHM smoothing kernel of 5 mm. Smoothing was masked by the respective participant’s brain mask provided by fMRIPrep.

In order to prepare data that were preprocessed in fMRIPrep for statistical analysis in FSL FEAT, it was necessary to perform a mock registration on the data in the “Preprocessing” section of FSL FEAT. When doing so, all preprocessing options were switched off with the exception of highpass temporal filtering (not an option in fMRIPrep) and registration to standard space (Linear, Normal search, 6 DOF). This step created the necessary file structure needed to run group-level statistical analysis of these data. Following this step, re-registered data for each participant were overwritten such that only registrations performed in fMRIPrep were taken forward for group-level statistical analysis^3^. Given that these steps are performed outside of fMRIPrep, estimated emissions, duration, and energy usage from this step were not included in the analysis presented in this paper. However, the computational resources required for this step were small (mean emissions = 0.00003 kg, mean duration = 0.017 hours, mean CPU energy = 0.00009 kWh, mean RAM energy = 0.000006 kWh). Their omission is unlikely to impact comparisons of preprocessing across packages.

Data for fMRIPrep were preprocessed in native resolution (in this case, 3 x 3 x 4mm), given that this is the default approach in fMRIPrep. Before appreciating that this is the default approach, we had initially intended to output data preprocessed in fMRIPrep to 2 mm standard space. The default approach was used to allow a fair comparison of default settings between packages. To quantify the effect of this deviation from our preregistration plan, we provide a comparison of all dependent variables for data preprocessed in fMRIPrep using either native or 2 mm resolution, in the Supplementary Section “Effects of fMRIPrep output space resolution” (Supplementary Fig. 1;Supplementary Table 2). In short, using 2 mm resolution increased the estimated carbon emissions of fMRIPrep by 44% and more than doubled the subsequent file size, with a modest but significant 6% increase in sensitivity to task activation.

#### 2.4.3 Model specification

For statistical analysis of preprocessed data, four regressors were created:

1. Go—Trials in the “go” condition for which participants responded correctly, with a fixed duration of 1.5 seconds.
2. Successful stop—Trials in the “stop” condition for which participants successfully inhibited their motor response, with a fixed duration of 1.5 seconds.
3. Unsuccessful stop—Trials in the “stop” condition for which participants did not successfully inhibit their motor response, with a fixed duration of 1.5 seconds.
4. Erroneous—Trials in the “go” condition for which participants responded incorrectly, with a fixed duration of 1.5 seconds. Ninety-nine participants had no erroneous “go” trials. In these cases, task data were modelled with 3 EVs, with “erroneous” omitted.

The onset time of each regressor was set as the start of the 0.5 second fixation that preceded each decision period^4^. Time periods not covered by the EVs include null periods between trials, and “go” trials on which participants provided no response (only 76 participants had any instance of this).

As inEsteban et al. (2019), we extracted the contrast of “go > successful stop,” producing a statistical image in which positive values reflected voxels activated by motor response, and negative values reflected voxels activated by successful response inhibition. The reverse of this contrast was also extracted to provide “successful stop > go.”

We had intended to include response time during “go” and “unsuccessful stop” trials as parametric regressors, orthogonalised to the respective fixed duration regressor. We opted not to do so given differences in handling of orthogonalisation in FSL FEAT and SPM (Mumford et al., 2015) that may complicate statistical comparison between packages. This analysis is reported in full in the Supplementary Section “Analysis with parametric regressors,” demonstrating dampened statistical effects in data analysed in FSL more so than in SPM, when including these parametric regressors (seeSupplementary Fig. 2;Supplementary Tables 3, 4).

#### 2.4.4 fMRI statistical analysis

Again, data preprocessed for all 248 participants in each package were statistically analysed separately, using general linear modelling (GLM). The specific steps implemented in each package are documented inTable 1and are summarised briefly below.

For data preprocessed in FSL BET and FEAT, first-level statistical analysis was also run in FEAT, set in the GUI to “Statistics” (rather than “Full analysis”). Settings were largely kept at default, with the exception that “Standard Motion Parameters” were added in the Stats tab. Again, for group-level statistical analysis of these data in FSL FEAT, settings were kept at default.

Data preprocessed in SPM were also subjected to first-level statistical analysis in SPM, again using multiple modules within a batch. Settings were largely kept at default, as determined in the SPM GUI. During fMRI model specification, the realignment parameters file generated during preprocessing was provided to the “Multiple regressors” field. Again, group-level statistical analysis for these data was conducted within SPM.

fMRIPrep is designed only to preprocess fMRI data, not for statistical analysis. As such, data preprocessed in fMRIPrep were statistically analysed in FSL FEAT. The same settings were used as for data preprocessed in FSL, with the exception that rather than using “Standard motion parameters” generated by FSL FEAT preprocessing, we used “Additional confound EVs” provided by fMRIPrep, relating to variables trans_x, trans_y, trans_z, rot_x, rot_y, and rot_z. Group-level statistical analysis for these data was also performed in FSL FEAT, using the same settings as for data preprocessed in FSL.

#### 2.4.5 Carbon tracking

All preprocessing and statistical analysis were run on the University of Sussex Apollo2 high-performance cluster, comprising 1,250 CPU cores across approximately 90 nodes. All tasks were run with five parallel environments/slots, on nodes using an Intel^®^Xeon^®^Processor E5-2640 v3. To estimate carbon emissions, we used an in-house tracking system that relies on retrospective use of HPC scheduler logs, developed for a previous study tracking the footprint of fMRIPrep variants (Souter et al., 2024). Energy usage (kWh) for CPU was estimated by multiplying total CPU time by the power usage of a CPU core. The power usage of a CPU core was obtained by dividing the thermal design power (TDP; 90 W) of the node in use, by the total number of available CPU cores (16). Energy usage for RAM (kWh) was estimated by multiplying maximum memory used (rounded up to the nearest GB) by volatile memory consumption (0.3725) and runtime. Estimated emissions (gCO_2_/kWh) were then calculated by multiplying the estimated energy usage by 207.07, the average carbon intensity value for the UK provided in the 2023 v1.1 release ofInternational Electricity Factors (2023)fromwww.carbonfootprint.com^5^, and by 1.28, the estimated power use effectiveness (PUE) of the Sussex HPC architecture as determined by data centre baseline power readings measured on October 19th 2023. This approach is analogous to that used by the Green Algorithms server-side tool GA4HPC (https://www.green-algorithms.org/GA4HPC). We extracted estimated emissions (kgCO_2_eq), duration of preprocessing (hours), and energy used by CPU and RAM (kWh).

As we used an average carbon intensity value, reported carbon emissions are proportional to total energy usage. We nevertheless report both estimated emissions and energy usage in order to investigate dissociable effects of software package and processing stage on CPU and RAM energy usage.

To facilitate separate carbon tracking, preprocessing and statistical analysis of data for a given participant in a given package were executed as separate tasks. Note that estimates of the footprint of statistical analysis for fMRIPrep will merely reflect the footprint of analysing data preprocessed in fMRIPrep within FSL FEAT, given that fMRIPrep does not include a statistical analysis function.

Carbon emissions were also estimated for ROI analysis in FSL Featquery (seesection 2.4.6below) for each participant. However, these estimates were not included in analysis of emissions between packages, given that ROI analysis is not strictly a part of first-level statistical modelling. It was not possible to track emissions resulting from smoothing in AFNI (as used for fMRIPrep data), given that AFNI is not currently set up to run on our HPC cluster. As such, the preprocessing emissions estimate for fMRIPrep does not account for smoothing. Carbon emissions were estimated for group-level statistical analysis of data for each package.

We further measured the average size (GB) of files generated for individual-level preprocessing and statistical analysis for each participant for each package, as well as the size of files generated during group-level statistical analysis.

#### 2.4.6 ROI analysis in FSL Featquery

To quantify preprocessing performance, we measured sensitivity to statistical activation in key regions of interest (ROIs) for each participant following first-level statistical analysis. Given that this was a key outcome measure for this study, we used a single tool to extract this information from data preprocessed/analysed in each package—FSL Featquery.

Regions were taken from the 400-network parcellation derived from resting-state and task data fromKong et al. (2021)—an update of parcellations bySchaefer et al. (2018). These parcellations are on GitHub (https://github.com/ThomasYeoLab/CBIG/tree/master/stable_projects/brain_parcellation/Schaefer2018_LocalGlobal/Parcellations/MNI), in the file “Schaefer2018_400Parcels_Kong2022_17Networks_order_FSLMNI152_2mm.nii.gz.” Regions were chosen according to their relevance to the stop-signal task. We identified which of the 400 parcellations best aligned with each expected site of activation.

For motor activation in the contrast of “go > successful stop,” an ROI in the left primary motor cortex was selected. For the contrast of “successful stop > go,” we selected two regions to reflect response inhibition activation, in the right pre-supplementary motor area (pre-SMA) and right insula (Rae et al., 2015;Sebastian et al., 2013;Zhang et al., 2017), and one to reflect exposure to the auditory “stop” stimulus, in the left auditory cortex. For each package, we extracted the mean t-statistic value^6^of voxels within each region for the relevant contrast (“go > successful stop” for primary motor cortex, “successful stop > go” for pre-SMA, auditory cortex, and insula). Higher mean t-statistics, as a proxy for sensitivity to statistical activation, were taken to reflect better performance. The four ROIs are visualised inFigure 1. The numeric ID and descriptive label of each region fromKong et al. (2021), as well as its size in voxels, are reported inTable 2.

**Fig. 1.**
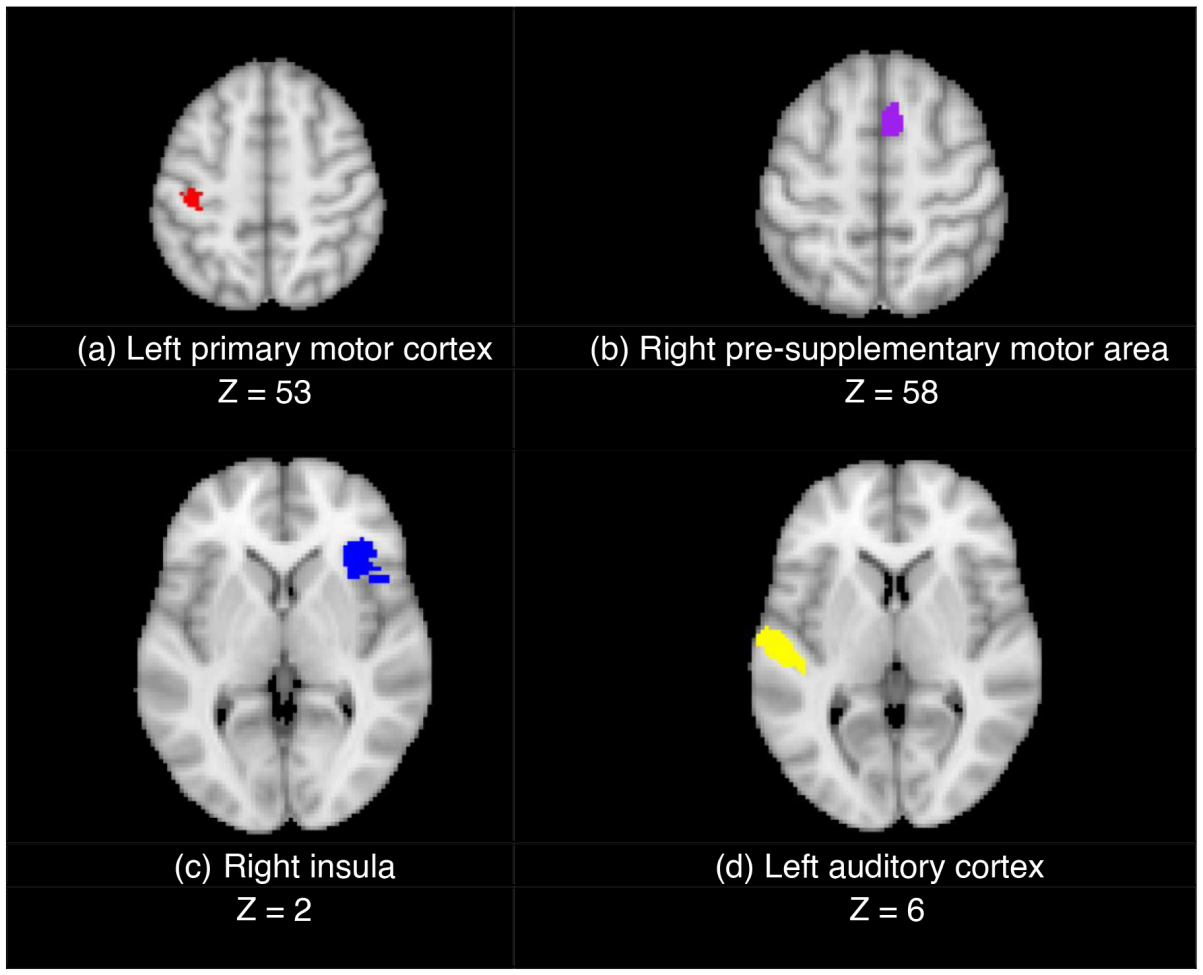
Regions of interest analysed in FSL Featquery, including (a) the left primary motor cortex (red) for “go > successful stop,” and the (b) right pre-supplementary motor area (purple), (c) right insula (blue), and (d) left auditory cortex (yellow) for “successful stop > go.” Regions are taken from a 400-region parcellation based on resting-state and task fMRI data fromKong et al. (2021); seeTable 2for details.

**Table 2.** Numeric ID and descriptive label for each region of interest, as taken fromKong et al. (2021), and size in voxels.

Region	Numeric ID	Label	Size (voxels)
Left primary motor cortex	151	17networks_LH_SomMotA_4	125
Right pre-supplementary motor area	286	17networks_RH_SalVenAttnA_FrMed_2	205
Left auditory cortex	141	17networks_LH_Aud_ST_3	490
Right insula	302	17networks_RH_SalVenAttnB_Ins_1	430

When running Featquery on data generated through SPM, it was necessary to register all ROI files to fit the same boundary box as SPM files. This was done using the FSL FLIRT function. This process in no way changed the location, shape, or size of the ROIs themselves. It was also necessary to set up the folder structure and filenames of relevant SPM files such that they would be judged as valid by FSL Featquery (for code to achieve this, see our repository on GitHub;https://github.com/NickESouter/fMRI-Computing-Footprint).

When running Featquery on data preprocessed in fMRIPrep, it was necessary to transform all ROI files to the native resolution of the input data and the standard output space generated in fMRIPrep by default (MNI152NLin2009cAsym). This was done using ANTs (see GitHub readme file for code;https://github.com/NickESouter/fMRI-Computing-Footprint). As outlined in the FEAT user guide, Featquery typically identifies the space of the input data and transforms ROI masks to fit it (https://fsl.fmrib.ox.ac.uk/fsl/fslwiki/FEAT/UserGuide#Featquery_-_FEAT_Results_Interrogation). For data preprocessed in FSL, masks would have been transformed into participants’ native space (given that standard space registration is not applied until higher-level statistical analysis) using FLIRT. The choice was made to use ANTs to transform ROIs for data preprocessed in fMRIPrep given that this is more in line with the methodology used in fMRIPrep and because, unlike data preprocessed in FSL FEAT, fMRIPrep derived data had already been registered to a shared standard space.

The change in total volume of each ROI as a result of ANTs transformation was calculated by multiplying the number of voxels in the original and transformed ROIs by the cubed resolution of the respective file size (native resolution—3 x 3 x 4 = 36; standard resolution—2 x 2 x 2 = 8). As seen inSupplementary Table 5, transformation in ANTs did result in a change in volume for each ROI (motor = -7.69%, pre-SMA = + 15.51%, auditory = -17.67%, insula = -6.52%). Use of subject-specific FLIRT transformation in Featquery would result in variation in exact ROI volume in any case. As seen inSupplementary Figure 3, the transformed regions are highly anatomically similar to the original ROIs. While ANTs transformation may mean that ROIs used for interrogation in fMRIPrep are larger or smaller around the peripheries of the regions, we do not anticipate that this would substantially bias comparisons of statistical task activation in these regions between fMRIPrep and other packages, given the volume changes were all less than 20%.

#### 2.4.7 Mean smoothness estimation in AFNI

As a second measure of preprocessing performance, the mean smoothness of data preprocessed in each package is reported in the Supplementary Section “Smoothness estimation” (Supplementary Figs. 4, 5). We preregistered use of this metric as a measure of preprocessing performance given that it had been used as such byEsteban et al. (2019)when comparing the performance of fMRIPrep and FSL, where larger mean smoothness values were taken as reflecting poorer pipeline performance, on the basis that increased smoothness reflects loss of anatomical specificity and statistical power. We have opted to move analysis of this variable to the supplementary materials given that, on reflection, improved signal-to-noise ratio as a result of smoothness may outweigh the downsides of decreased spatial resolution (seeCaballero-Gaudes & Reynolds, 2017;Triantafyllou et al., 2006), and statistical comparison of smoothness estimates may be influenced by differences in the underlying resolution of the data for each package. In the current context, this metric is, therefore, likely not as valuable a performance metric as statistical task activation. In short, mean smoothness was higher for fMRIPrep than FSL (1.1x) and SPM (1.6x), and for FSL than SPM (1.4x).

A full summary of the experimental procedure is provided inFigure 2.

**Fig. 2.**
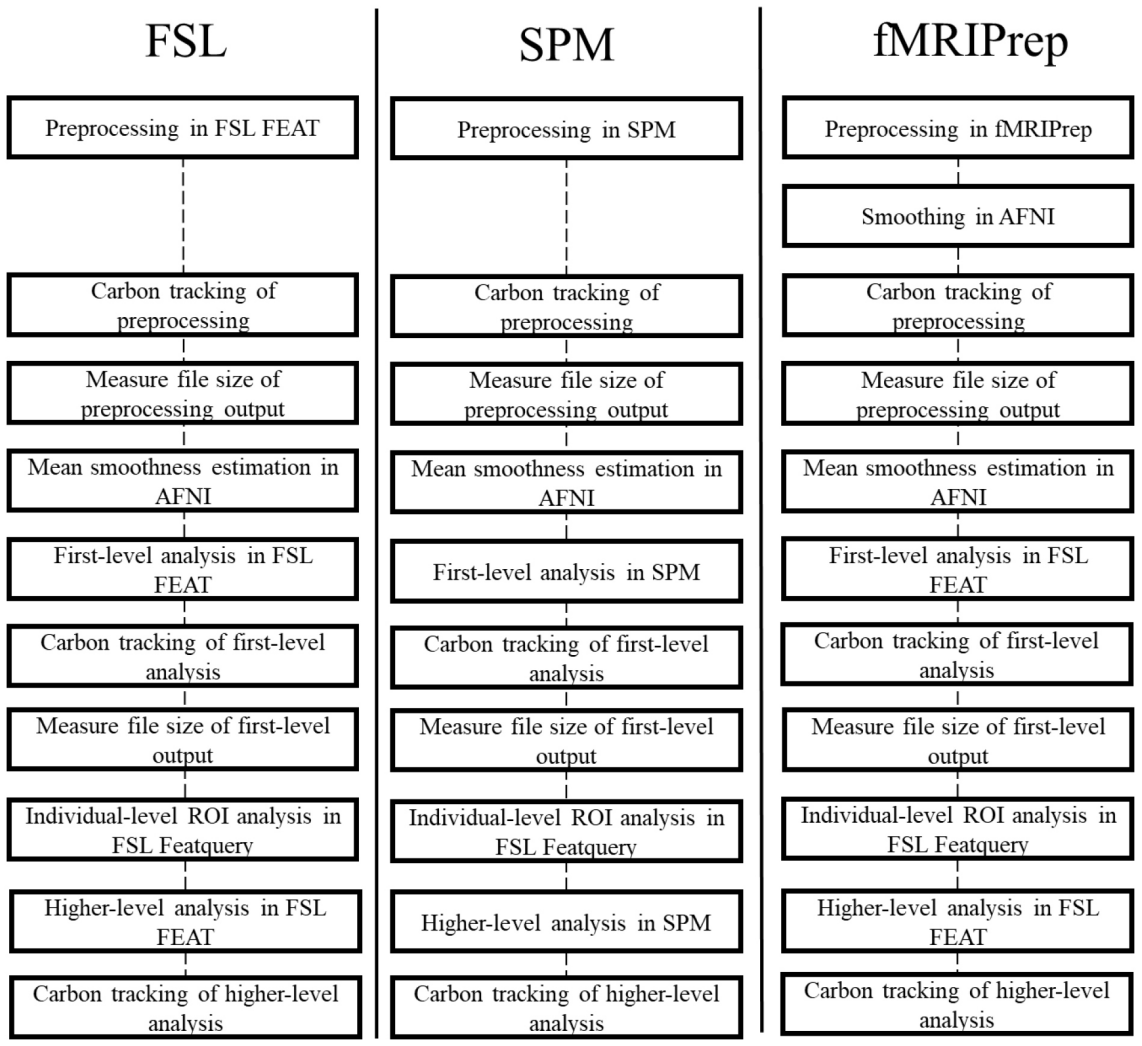
Experimental procedure of preprocessing and statistical analysis steps, with outcome measures derived from each stage. CPU = central processing unit, RAM = random access memory, GLM = generalised linear model.

### 2.5 Data analysis

To summarise, dependent variables include the computing metrics (1) estimated carbon emissions (kgCO_2_eq), (2) energy (kWh) for CPU and RAM, (3) duration (hours), and (4) file size (GB), all split by preprocessing and statistical analysis, and (5) task activation (t-statistic) in four ROIs. Prior to analysis, outliers (greater than 3 standard deviations above or below the group mean) for each level of the respective variable for the respective package were removed.

Repeated measures ANOVAs were used to test for effects of package on each dependent variable. When possible, we tested main effects and interactions of stage, energy source, and region. When significant effects or interactions were observed, ANOVAs were followed by Wilcoxon tests comparing this variable across all relevant levels (includes Package [FSL, SPM, fMRIPrep], Stage [preprocessing, statistical analysis], and Source [CPU, RAM]). For each set of contrasts for a given effect or interaction, we applied false discovery rate (FDR) correction using the Benjamini–Hochberg method. All ANOVAs and contrasts were run in R (version 4.2.2) using the rstatix package (version 0.7.2;https://rpkgs.datanovia.com/rstatix).

We next compared thresholded group-level activation for data preprocessed in each package, for the contrasts of “go > successful stop” and “successful stop > go.” Images used for plotting neuroimaging data in the current paper are reproducible in R (seehttps://osf.io/cdq6y), using guidance fromChopra et al. (2023). Finally, Bland–Altman plots were used to plot, for each pair of unthresholded group-level maps, the relationship between the mean of and difference between values for a given voxel. This should provide a reflection of the heterogeneity of data derived from each package. For both group-level comparisons, the boundary box of group-level output for data analysed in SPM was adjusted to be the same size as that of data analysed in FSL FEAT, using the FSL FLIRT function.

Note that in order to compare group-level output across packages, it was necessary to transform output generated for fMRIPrep preprocessed data (in MNI152NLin2009cAsym) into the template space shared by both FSL and SPM (MNI152NLin6Asym), using ANTs (antsApplyTransforms), both for unthresholded and thresholded statistical maps. For thresholded output files used in overlaying group-level results, the files transformed were binarised before transformation. Transformation caused minimal deviations in the shape of these binarised clusters. For the unthresholded map of “go > successful stop,” used for Bland–Altman plots, transformation did entail statistical values changing somewhat. However, the pattern and peaks of activation remained consistent. Unthresholded fMRIPrep maps both pre- and post-transformation are on Neurovault (https://neurovault.org/collections/QRJOSICN).

The results section finishes with contextualisation of the carbon footprint of fMRI data analysis in FSL, SPM, and fMRIPrep, relative to the activities of collecting fMRI data, commuting to work via petrol passenger car, and a return flight from London to New York City. The methods used to quantify these metrics are discussed when presenting these data.

The computational reproducibility of the results reported in this study has been checked by an independent statistician (seehttps://osf.io/npv2dfor the full report). This was conducted as part of the University of Sussex School of Psychology initiative “Ensuring the computational reproducibility of to-be-submitted psychology papers” (https://doi.org/10.17605/osf.io/dr35v).

## 3 Results

### 3.1 Package comparisons

**Fig. 3.**
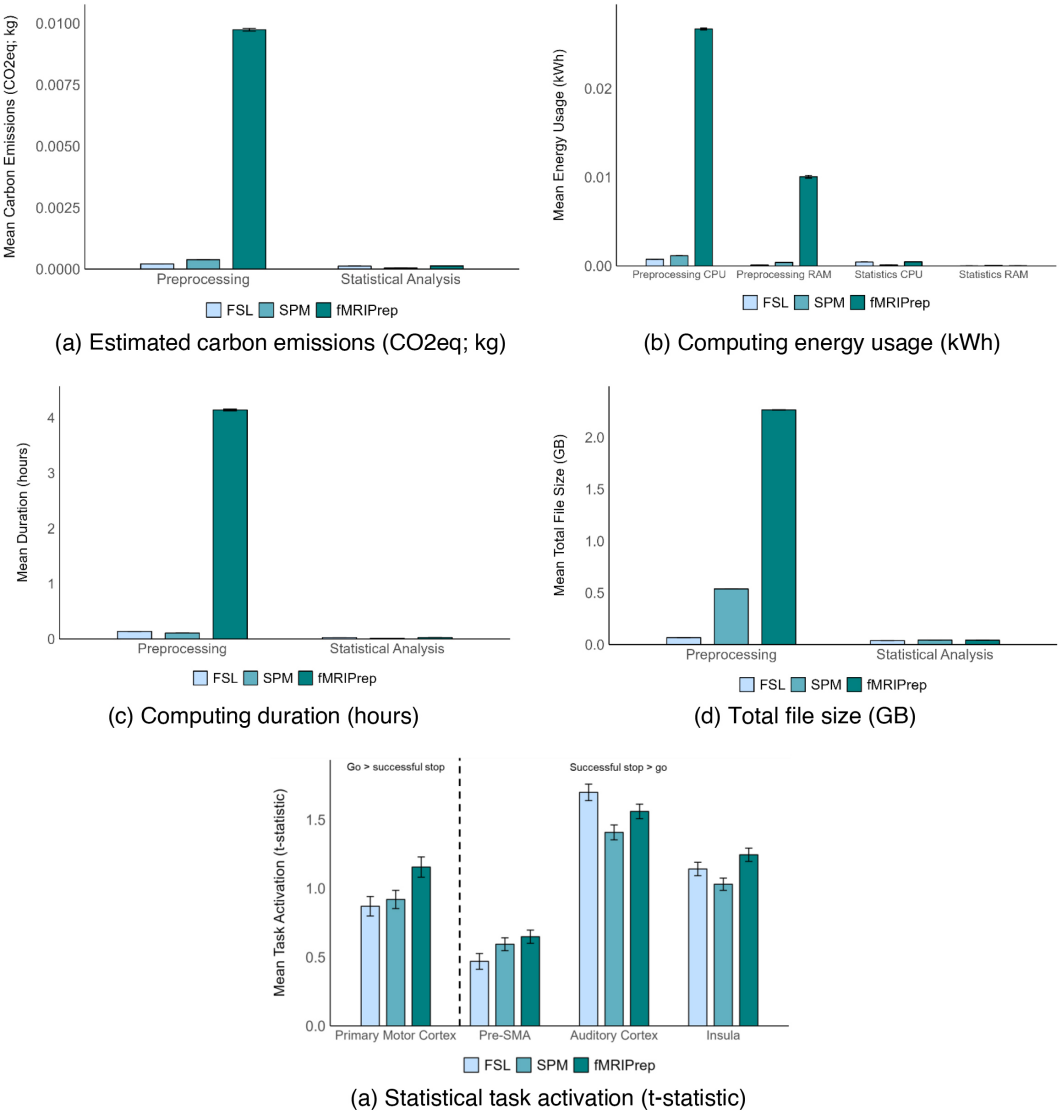
Mean values (per participant) for each package for (a) estimated carbon emissions, (b) energy usage, (c) duration of computing, (d) total file size, and (e) statistical activation in regions of interest (the dotted line in this plot separates regions according to the contrast for which they were interrogated: ‘Go > successful stop’ for Primary Motor Cortex; ‘Successful stop > go’ for Pre-SMA, Auditory Cortex, and Insula). Metrics split by stage (preprocessing/statistical analysis), energy source (CPU/RAM), and region of interest when appropriate. Error bars reflect one standard error of the mean. These are frequently too small to be visible. Pre-SMA = pre-supplementary motor area, CPU = central processing unit, RAM = random-access memory, CO_2_eq = carbon dioxide equivalent, kWh = kilowatt hours, kg = kilograms.

Figure 3presents mean values (per participant) for each dependent variable for each package (FSL/SPM/fMRIPrep). When appropriate, metrics are split by stage (preprocessing/statistical analysis), energy source (CPU/RAM), and region (primary motor cortex/pre-SMA/auditory cortex/insula).

Repeated measures ANOVAs are reported inTable 3. All dependent variables were significantly influenced by each of the relevant independent variables. Most notably, the estimated energy usage (and, therefore, carbon emissions) and file size for fMRIPrep were substantially greater than for FSL and SPM, while also providing evidence of somewhat greater task activation in ROIs.

**Table 3.** Repeated measures ANOVAs for carbon emissions, energy usage, duration, total file size, and task activation.

Variable	Effect	RESULT
Carbon emissions	Package ^a^	F(1.0, 233.6) = 28,617.1, *p* < .001*, η _p_^2^ = .99
	Stage	F(1, 233) = 32,074, *p* < .001*, η _p_^2^ = .99
	Package x stage ^a^	F(1.0, 233.5) = 27,983, *p* < .001*, η _p_^2^ = .99
Energy usage	Package ^a^	F(1.0, 222.9) = 38,627.8, *p* < .001*, η _p_^2^ = .99
	Stage	F(1, 222) = 43,746.0, *p* < .001*, η _p_^2^ = .995
	Source	F(1, 222) = 11,269.6, *p* < .001*, η _p_^2^ = .98
	Package x stage ^a^	F(1.0, 222.7) = 37,707.2, *p* < .001*, η _p_^2^ = .99
	Package x source ^a^	F(1.0, 222.5) = 8,214.1, *p* < .001*, η _p_^2^ = .97
	Stage x source	F(1, 222) = 9,241.4, *p* < .001*, η _p_^2^ = .98
	Package x stage x source ^a^	F(1.0, 222.3) = 7,880.6, *p* < .001*, η _p_^2^ = .97
Duration	Package ^a^	F(1.0, 236.1) = 60,346.2, *p* < .001*, η _p_^2^ = .996
	Stage	F(1, 235) = 68,895.2, *p* < .001*, η _p_^2^ = .997
	Package x stage ^a^	F(1.0, 235.9) = 60,025.9, *p* < .001*, η _p_^2^ = .996
Total file size	Package ^a^	F(1.1, 255.9) = 1,079,526.8, *p* < .001*, η _p_^2^ > .999
	Stage	F(1, 240) = 2,134,057.6, *p* < .001*, η _p_^2^ > .999
	Package x stage ^a^	F(1.0, 240.5) = 1,131,918.2, *p* < .001*, η _p_^2^ > .999
Task activation	Package ^a^	F(1.9, 446.5) = 48.1, *p* < .001*, η _p_^2^ = .17
	Region ^a^	F(2.0, 481.6) = 58.4, *p* < .001*, η _p_^2^ = .20
	Package x region ^a^	F(4.8, 1,153.1) = 23.8, *p* < .001*, η _p_^2^ = .09

Note: *Reflects significant results at*p*< .05.

a Reflects Greenhouse–Geisser correction applied due to violation of the assumption of sphericity. Levels for effects are as follows: Package (FSL, SPM, fMRIPrep), Stage (Preprocessing, Statistical analysis), Source (CPU, RAM), Region (Primary motor cortex, Pre-supplementary motor area, Auditory cortex, Insula).

Effects and interactions for each dependent variable are parsed below. All effects are discussed except the main effect of*Region*for Task activation, which is not of experimental interest given that comparisons of activation between regions would not provide further insight into effects of package on scientific performance. All post hoc inference is based on planned contrasts with false discovery rate correction using the Benjamini–Hochberg method (Supplementary Table 6). Computing metrics for group-level statistical analysis are inSupplementary Table 7. While direct statistical comparison across packages for this group-level data was not possible (due to there only being one observation per package), descriptive comparisons are provided.

#### 3.1.1 Carbon emissions

Total estimated carbon emissions for fMRIPrep were 30 times (30x) higher than for FSL, and 23x higher than for SPM. Emissions for SPM were 1.3x higher than for FSL. Across packages, emissions were higher for preprocessing than for statistical analysis, with this difference being largest for fMRIPrep (76x), followed by SPM (8.7x), and then FSL (1.7x).

For group-level statistical analysis, estimated carbon emissions were 0.02640 kgCO_2_e for FSL, 0.00498 kgCO_2_e for fMRIPrep, and 0.00007 kgCO_2_e for SPM. The footprint for SPM is far lower than either other package, likely because in FSL FEAT registration to standard space and resolution is applied at the group level, while in SPM, this is done at the individual level. The footprint for FSL is larger than fMRIPrep, likely because individual data preprocessed in fMRIPrep are already in standard space, while data for FSL are in native space. This same explanation likely accounts for similar differences in energy usage, duration, and file size below.

#### 3.1.2 Energy usage

Mirroring carbon emissions, overall energy usage for fMRIPrep was 29x greater than FSL and 22x greater than SPM. Energy usage for SPM was 1.3x greater than for FSL. There was higher average energy usage for preprocessing than statistical analysis, with this difference being largest for fMRIPrep (76x), followed by SPM (9.3x), and then FSL (1.9x).

There was higher average energy usage for CPU than for RAM. The magnitude of this effect also varied by package, although the absolute difference between sources deviated from proportional difference; FSL (11x; 0.00054 kWh), SPM (2.8x; 0.00041 kWh), fMRIPrep (2.7x; 0.00855 kWh). Difference in CPU and RAM similarly varied by stage, again with deviations in absolute and proportional difference: preprocessing (2.7x; 0.00602 kWh), statistical analysis (12x; 0.00031 kWh). As demonstrated by an interaction of package, stage, and source, energy usage was consistently significantly greater for CPU than for RAM, with the extent of this difference varying by package and stage; FSL preprocessing (7.5x, 0.00065 kWh), FSL stats (44x, 0.00043 kWh), SPM preprocessing (2.9x, 0.00076 kWh), SPM stats (2.0x, 0.00005 kWh), fMRIPrep preprocessing (2.7x, 0.01665 kWh), fMRIPrep stats (23x, 0.00044 kWh).

Energy usage for group-level statistical analysis was 0.0996 kWh for FSL (CPU = 0.0941; RAM = 0.0055), 0.0188 kWh for fMRIPrep (CPU = 0.0178; RAM = 0.0010), and 0.0003 kWh for SPM (CPU = 0.0002; RAM = 0.0001).

#### 3.1.3 Duration

Total duration of computing for fMRIPrep was 26x longer than FSL and 34x longer than SPM. Duration for FSL was 1.3x longer than SPM. Across packages, the duration of preprocessing was longer than that of statistical analysis, with this difference being largest for fMRIPrep (150x), followed by SPM (7.1x), and then FSL (5.1x).

The duration of group-level statistical analysis was 4.90 hours for FSL, 1.34 hours for fMRIPrep, and .03 hours (2.1 minutes) for SPM.

#### 3.1.4 Total file size

Average total file size was larger for fMRIPrep than for FSL (22x) and SPM (4.0x). File size for SPM was larger than for FSL (5.4x). Size was larger for files generated during preprocessing than during statistical analysis, with this difference being greatest for fMRIPrep (53x), followed by SPM (12x), and then FSL (1.7x). The size of files generated by fMRIPrep can be reduced up to 96% by cleaning up working directory files and unneeded derivatives (Souter et al., 2023).

For group-level statistical analysis, total file size was 4.68 GB for FSL, 0.84 GB for fMRIPrep, and 0.04 GB for SPM.

#### 3.1.5 Task activation

Across ROIs, mean statistical activation was greater for fMRIPrep than for FSL (1.1x) and SPM (1.2x), and for FSL than for SPM (1.1x). This pattern varied by region. For the primary motor cortex, activation was greater for fMRIPrep than for FSL (1.3x) and SPM (1.3x), with no significant difference between FSL and SPM. For the pre-SMA, activation was greater for fMRIPrep than for FSL (1.4x) and SPM (1.1x), and for SPM than for FSL (1.3x). For the auditory cortex, activation was greater for FSL than for SPM (1.2x) and fMRIPrep (1.1x), and for fMRIPrep than for SPM (1.1x). In the insula, activation was greater for fMRIPrep than for FSL (1.1x) and SPM (1.2x), and for FSL than for SPM (1.1x).

### 3.2 Comparison of group-level output

**Fig. 4.**
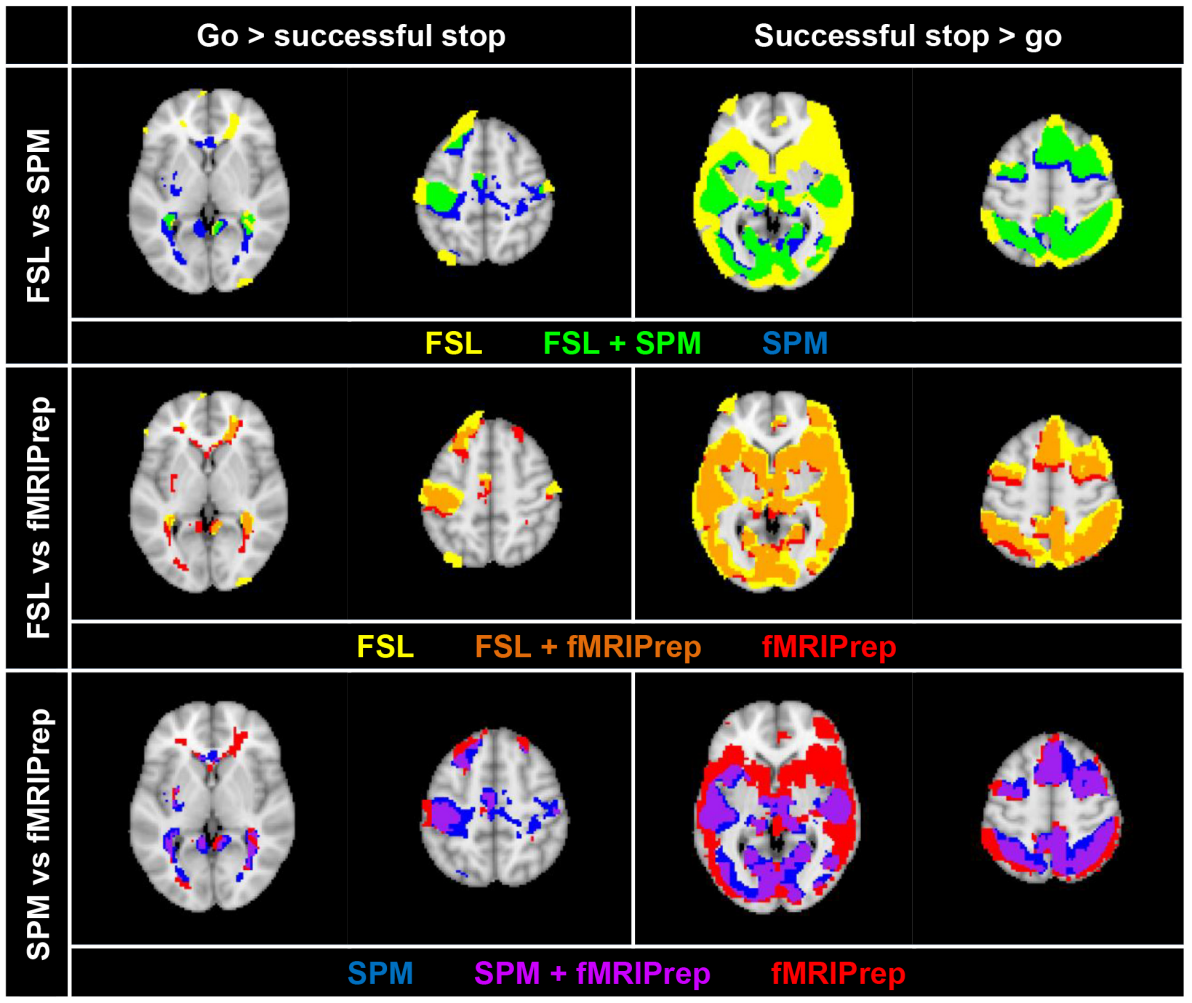
Overlap in thresholded (*p*< 0.05 FWEc) group-level statistical activation for each combination of packages, for the contrasts of “go > successful stop” and “successful stop > go.” Colours reflect the package (or overlap of packages) for which thresholded activation is plotted in a given panel. Generated in R using the neurobase package. Slices presented are at MNI coordinates Z= 4 (left) and Z = 52 (right).

Figure 4presents overlapping clusters for the thresholded (*p*< 0.05 FWEc) contrasts of “go > successful stop” and “successful stop > go” from group-level statistical analysis, for each combination of packages. Unthresholded group-level maps are on Neurovault (https://neurovault.org/collections/QRJOSICN). The size and relative overlap for both contrasts for each package (as measured by dice coefficients^7^) are given inSupplementary Table 8. The four regions used as ROIs were all represented in each package’s group-level map for the relevant contrasts. Despite this, there was heterogeneity in the number and anatomical distribution of activated voxels across packages. For example, for “go > successful stop,” agreement with FSL output was only 47% for SPM and 52% for fMRIPrep, as measured by dice coefficients. This may be accounted for by the fact that FSL clusters frequently included voxels outside the brain mask—this was uncommon for SPM and fMRIPrep. This may suggest poorer spatial precision for data preprocessed in FSL, possibly attributable to artefacts generated during skull stripping. However, this disparity is also likely due in part to differences in brain masking procedures between software packages, given that by default, brain masking is not performed in higher-level analysis in FSL.

**Fig. 5.**
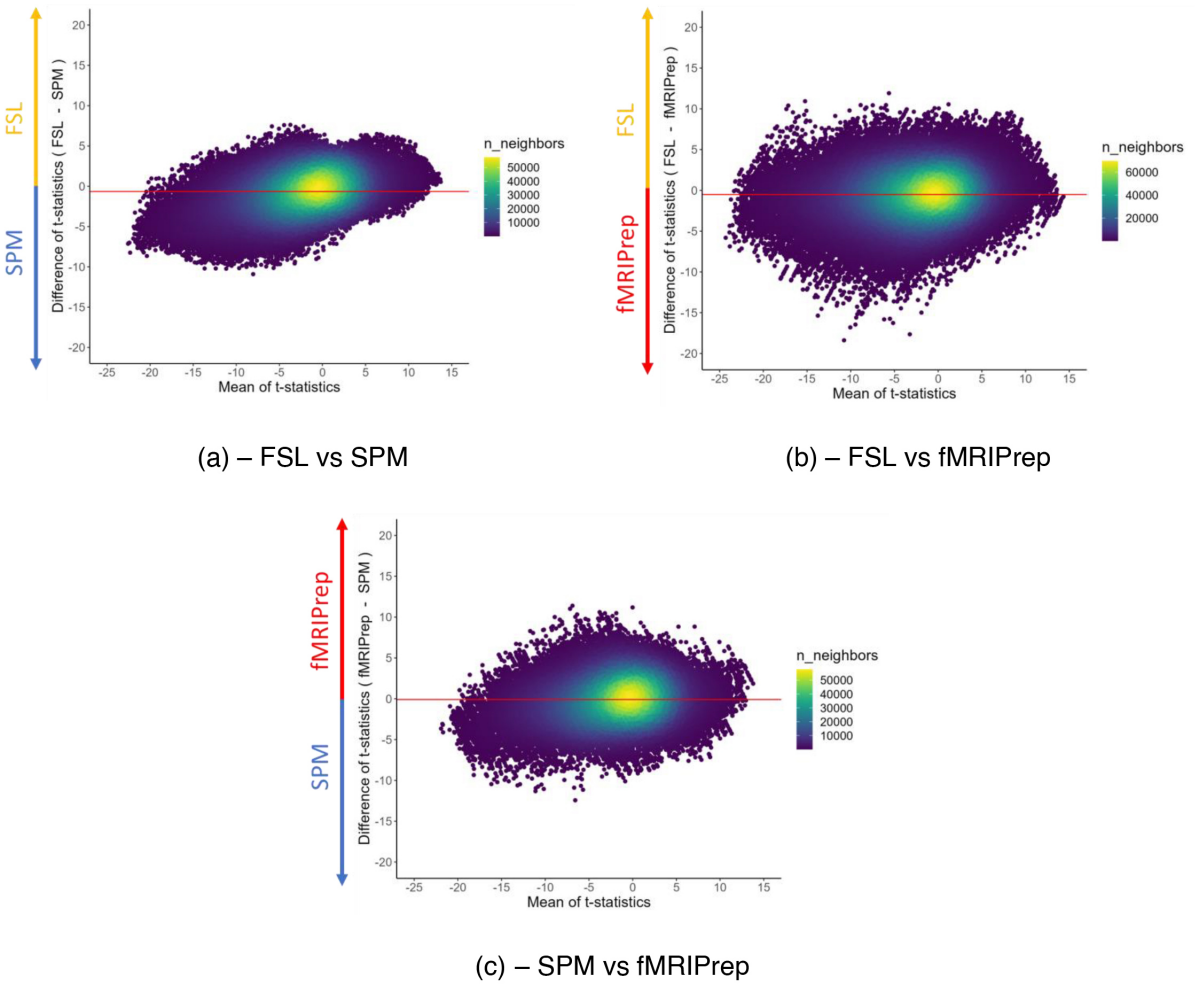
Bland–Altman plots comparing unthresholded group-level maps for data processed in each package. This includes a comparison of (a) FSL and SPM, (b) FSL and fMRIPrep, and (c) SPM and fMRIPrep. Voxels represented are in the intersection of masks of non-zero voxels for the relevant combination of packages. n_neighbors = number of neighbours. Created using the ggpointdensity package (Kremer & Anders, 2019). Red horizontal line reflects the mean difference value for a given comparison.

Next, Bland–Altman plots (seeFig. 5) represent the extent of statistical agreement in unthresholded group-level maps for the contrast of “go > successful stop,” following the procedure used byBowring et al. (2019). For the comparison of two packages (e.g., FSL and SPM), the mean t-statistic in each non-zero voxel is plotted against the difference between these same two values. Several inferences can be made. First, the difference between packages appears greatest when comparing FSL and fMRIPrep, followed by SPM and fMRIPrep, and then FSL and SPM. Second, for each comparison, the density of voxels is highest at the point at which both the difference between and the mean of values is close to 0, reflecting relatively mild statistical effects that are homogeneous across packages. This is supported by the fact that each mean difference between packages (red line on each plot) is close to zero. Third, for each comparison, a larger number of voxels have a mean negative value (left of zero on the x-axis) than positive, implying that overlapping statistical values are greater for the contrast of “successful stop > go” than “go > successful stop.” This is consistent with the fact that thresholded group-level maps for “successful stop > go” are larger in size than the inverse for each package (FSL = 3.7x, SPM = 2.3x, fMRIPrep = 3.7x;Supplementary Table 8). This may reflect more widespread neural engagement required for successful motor inhibition than for button pressing. Finally, voxels which have a larger statistical value for SPM compared with FSL (Fig. 5a) tend to sit more towards the “successful stop” end of the contrast than “go.” This is also true for voxels which have a larger value for SPM than fMRIPrep (Fig. 5c). Conversely, voxels which have a larger value for FSL than fMRIPrep (Fig. 5b) tend to sit more towards the “go” end of the contrast than “successful stop.” Overall, despite qualitative similarities, there is deviation across packages.

### 3.3 Contextualisation of carbon emissions

Finally, we provide contextualisation of the scale of carbon emissions used for fMRI data analysis relative to fMRI data collection, commuting in a petrol passenger car, and a return trans-Atlantic flight from London to New York City.

To quantify the emissions of fMRI analysis, estimates were derived from the combination of preprocessing, first-level statistical analysis, Featquery, and group-level statistical analysis for all 248 participants for the current study, for each package.

For fMRI data collection, the estimate is based on data fromChodorowski et al. (2024)where 4m 12s of fMRI echo planar imaging (EPI) was found to have energy usage of 3.25 kWh. This energy usage was scaled up to 6 minutes (the approximate duration of the stop signal task used for the current study), amounting to 4.64 kWh. This was then applied to a sample of 248 participants, equalling 1,151.43 kWh. This was multiplied by 207.7, the carbon intensity value used for the current study, and then converted from grams to kg for the final estimate.

For commuting by car, the carbon emissions value provided is based on the Mobilityways Commuter Census 2024 (https://mobilityways.com/2024-commuter-census-an-executive-summary), which found the average distance for UK single occupancy vehicle commuters to be approximately 19.5 miles. This was doubled to 39 miles (62.76 km) to account for a return journey. This was multiplied by a value of 108.1 gCO_2_/km, as taken from the 2022 average estimate of new passenger cars in Europe from the European Environment Agency (https://www.eea.europa.eu/en/analysis/indicators/co2-performance-of-new-passenger), and converted to kg for the provided estimate.

For the return trans-Atlantic flight, the Travel Carbon Footprint Calculator (https://travel-footprint-calculator.irap.omp.eu/) was used to estimate the carbon emissions associated with a return flight from London to New York City (11,140 km; 6,922 miles) on a passenger plane.

The resulting comparisons are given inTable 4. Further reflection on these comparisons is provided below in the Discussion section.

**Table 4.** Estimated energy usage (kWh) and carbon emissions (kgCO_2_e) for fMRI data analysis, fMRI data collection, return commute via car, and a return trans-Atlantic flight.

Activity	Notes	Energy usage (kWh)	Carbon emissions (kgCO _2_ e)
fMRI analysis in FSL	Preprocessing and statistical analysis in FSL FEAT, 6 minutes of fMRI data for 248 participants, based on current results.	0.46	0.12
fMRI analysis in SPM	Preprocessing and statistical analysis in SPM12, 6 minutes of fMRI data for 248 participants, based on current results.	0.44	0.11
fMRI analysis in fMRIPrep	Preprocessing in fMRIPrep and statistical analysis in FSL FEAT, 6 minutes of fMRI data for 248 participants, based on current results.	9.51	2.52
fMRI data collection	Estimate for 6 minutes of fMRI EPI data collection for 248 participants, based on reporting by Chodorowski et al. (2024)	1,151.43	239.15
Return commute to work by petrol car (38 miles)	Assuming a commuting distance of 19.5 miles (31.4 km), the average value reported for the UK in the Mobilityways Commuter Census 2024, and a carbon conversion factor of 108.1 gCO _2_ /km as taken from the European Environment Agency.	N/A	6.78
Return trans-Atlantic flight	Return flight from London to New York City, calculated using the Travel Carbon Footprint Calculator	N/A	1,917.58

Note: fMRI = functional magnetic resonance image, FSL = FMRIB Software Library, FEAT = fMRI Expert Analysis Tool, SPM = Statistical Parametric Mapping, EPI = echo-planar imaging, gCO_2_/km = grams of carbon dioxide per kilometre, kWh = kilowatt hours, kgCO_2_e = kilograms of carbon dioxide equivalent emissions.

## 4 Discussion

We measured and compared the estimated carbon emissions of analysing fMRI data in FSL, SPM, and fMRIPrep. On average, for preprocessing and statistical analysis of 6 minutes of task-fMRI for a single participant, use of fMRIPrep (including preprocessing in fMRIPrep and statistical analysis in FSL FEAT) emitted 0.0098 kg of CO_2_—30 times higher than in FSL (0.0003 kg) and 23 times higher than in SPM (0.0004 kg). Similarly, an average file size of 2.3 GB was generated by fMRIPrep, compared with 0.6 GB for SPM and only 0.1 GB for FSL. The bulk of emissions and files arose from preprocessing, rather than statistical analysis. To quantify scientific performance, we observed mean statistical activation. On average, fMRIPrep presented with the highest activation. This pattern varied by region—with the auditory cortex showing an advantage for FSL over fMRIPrep. While FSL presented with overall higher activation than SPM, this also varied by region, with equivalence between packages for the primary motor cortex and greater activation for SPM in the pre-SMA.

Use of fMRIPrep may produce substantial emissions when used with large datasets such as UK Biobank (Miller et al., 2016), or if in a location with high carbon intensity or inefficient hardware. The large relative difference in emissions between fMRIPrep and other packages FSL and SPM is likely attributable to the use of more contemporary but computationally intensive tools. For example, ANTs, a tool used at multiple stages in fMRIPrep, may provide maximally precise registration at the cost of increased compute time and, therefore, energy usage. This difference is likely also due in part to the use of containerisation (in this case, in singularity) for fMRIPrep, the use of which is likely to have overheads needed to create a standard and flexible environment. Indeed, prior work has suggested that containerisation affects the energy efficiency of computing (Santos et al., 2018). While fMRIPrep can be installed without containerisation, this is a technically demanding process that will not be employed by many users. Neuroimaging researchers may judge the increased scientific precision, and other specific advantages provided by fMRIPrep, to be worth the relative increase in carbon emissions. Particularly when investigating low powered effects, fMRIPrep may increase sensitivity to meaningful statistical activation, as well as providing increased anatomical specificity (as seen through qualitative comparison of group-level statistical maps). Furthermore, the preprocessing options included in and the computational resources allocated to fMRIPrep can be changed directly in the command line. This, in conjunction with the requirement of the standardised Brain Imaging Data Structure (BIDS) for input, may make it easier to automatically and reproducibly preprocess large datasets compared with other packages.

Although the estimated emissions and subsequent file size of fMRIPrep were substantially greater than for both FSL and SPM, differences were also found between these packages. Compared with FSL, SPM produced 30% greater estimated carbon emissions when processing individual-level data as well as producing over five times the subsequent file size. Despite this, FSL provided overall greater evidence of statistical activation. It may be possible for SPM to increase individual-level efficiency in future iterations by following the model of FSL—processing data in participants’ native space and only normalising the relevant derivatives. However, as seen inTable 4, the total difference in emissions between FSL and SPM disappears when considering the combination of individual-level and group-level analysis. Based on the current findings, both FSL and SPM are computationally lightweight in absolute terms, and can likely be used without the cost of substantial computing emissions.

Increasing confidence in evidence-based conclusions is a crucial feature of robust and worthwhile science. If use of best-practice tools coincides with substantial increases in carbon emissions, we suggest that researchers should not be dissuaded from making this choice. However, one should be mindful of how such tools are used, and adjust usage to minimise emissions where possible. For instance, as previously demonstrated (Souter et al., 2024), carbon emissions in fMRIPrep can be reduced by 48% by disabling FreeSurfer surface reconstruction (as was done in the current study) when the resulting files are not needed. In supplementary analysis for the current paper, we demonstrated that requesting fMRIPrep data in standard 2 mm resolution rather than default native resolution can increase carbon emissions by 44% and more than double file size, but with a modest increase in statistical sensitivity (an average of 6%).

Researchers can also adopt wider practices to reduce the footprint of their analysis, including scheduling tasks to run at times of low carbon intensity, when the relevant energy grid in use is relying maximally on renewable sources (see CATS;https://github.com/GreenScheduler/cats), and cleaning up junk files (see fMRIPrepCleanup;https://github.com/NickESouter/fMRIPrepCleanup;Souter et al., 2023, and SPMCleanup;https://github.com/NickESouter/SPMCleanup). Finally, researchers can be mindful of long-term storage for large datasets, both in terms of local servers and cloud computing services. For instance, publicly sharing preprocessed rather than raw fMRI data may aid in avoiding unnecessary duplication of preprocessing, and decrease strain on storage (Souter et al., 2023). By implementing green computing practices and raising awareness, researchers can also put pressure on institutions to invest more in efficient computing infrastructure and central resources (e.g., technical support) that would contribute to increasingly green computing.

Beyond the responsibilities of individual users, developers of analysis tools can take steps to ensure that required compute is minimised while still facilitating the production of high-quality output. For example, fMRIPrep could provide the option of adding “carbon-minimal flags” to users’ command lines (e.g., constraining memory usage and automatically deleting working directory files). Additions to recent fMRIPrep versions demonstrate positive steps, including the option to request varying levels of derivatives depending on one’s research needs (version 23.2.0) and to use previously generated derivatives when re-running preprocessing (version 25.0.0). Through such initiatives, developers can foster a culture of creating research tools that produce robust and easily reproducible results while also considering environmental sustainability. Tools with a smaller footprint, such as FSL and SPM, can further incentivise their own use by developing an easily reproducible and usable interface to match that of more recent command-line operated tools such as fMRIPrep.

Even when not using compute-intensive tools, one should not be complacent. Researchers can make use of carbon tracking tools (Lannelongue & Inouye, 2023) to provide an “Environmental impact statement” (Souter et al., 2023) in research papers, providing an overall estimation of carbon emissions generated through data processing. An example of such a statement can be seen at the end of this paper, indicating an overall footprint of 6.38 kg of carbon dioxide equivalent emissions for the current study. For studies with computing footprints of any size, doing so can help to socially norm paying attention to the environmental impact of our science. We can foster a social expectation akin to that of a “Data availability statement,” the introduction of which by journals has transformed research culture (Colavizza et al., 2020).

Beyond computing, researchers should be sensitive to other potentially carbon-intensive practices, and seek to reduce them.Table 4provides context on the extent of energy usage and estimated carbon emissions for the fMRI data analysis conducted for the current study. This comparison demonstrates that the carbon footprint of fMRI data processing and analysis is small relative to other scientific activities, with use of even fMRIPrep constituting just over 1% of the emissions associated with collecting the equivalent amount of fMRI data. In an even more stark comparison, total use of fMRIPrep for the current study would amount to just over 0.1% of the carbon emissions associated with a return flight from London to New York City, a journey that may be used by a researcher for conference travel. These data demonstrate the urgency of finding environmentally sustainable solutions to data collection, and to cutting emissions arising as a result of international conference travel. Indeed, flying less to international conferences may be the most impactful way for neuroscientists to reduce their research carbon footprint (Epp et al., 2023). Researchers organising workshops and conferences can also provide plant-based food, given that the environmental impact of animal products typically far exceeds that of vegetable protein alternatives (Poore & Nemecek, 2018). Given that “carbon offsets” do not currently provide a viable route to compensating for our effect on the climate (Watt, 2021), researchers should attend to, and seek to minimise, environmental impacts arising from their academic activity, wherever possible. Funding bodies can play a role here by requesting or requiring that applicants transparently report the environmental impact of their research, either prospectively or retrospectively (Rae et al., 2022). The Wellcome Trust provides an example of such an initiative^8^. This paper focused on data analysis given that this is one aspect of their footprint over which researchers have maximal control with minimal trade-offs. It is important to develop good practices in this area, given that the likely adoption of machine learning techniques in MRI research (e.g.,Cáceres et al., 2024;Nenning & Langs, 2022) may cause analysis to become increasingly energy intensive in coming years. While available renewable energy may be increasing in some countries, this availability is fundamentally limited by infrastructure capacity, which may struggle to keep pace with the rising energy demands of artificial intelligence and machine learning^9^.

There are limitations to the current study. First, there is no accepted best practice for quantifying fMRI data output quality. Methods such as split-half resampling (Churchill et al., 2015) may be viewed by some as preferable to statistical task activation. Task activation is limited in so far as different sets of confounds were used for each software package, meaning that inherently different effects may be captured (as reflected in variable patterns of software package on different regions of interest). However, largely default settings were used in analysis for each package, and as such the results reported should reflect the experience of the average user. Furthermore, there is no absolute ground truth in brain activation that a given task should evoke. In this sense, statistical activation is not a definitive measure of preprocessing performance, and a given package cannot be labelled as definitely and empirically better than another. These results should, therefore, be treated with caution. The issue could have been somewhat addressed by using a simulated dataset for which the “ideal” pattern of activation is known (e.g.,Durnez et al., 2016;Mumford et al., 2014;Welvaert & Rosseel, 2014). Doing so was beyond the scope of the current study, but future studies benchmarking carbon emissions and preprocessing performance may benefit from such an approach. Second, while methods of measuring computing energy usage have been validated (Jay et al., 2023), use of thermal design power as in the current study may underestimate usage (Lannelongue et al., 2023). Furthermore, our energy usage calculations did not account for idle power consumption of computing nodes, that is, power used by servers while not actively contributing to our jobs. Although this should be accounted in order to obtain a complete picture of total energy usage of a given task, idle power consumption is highly dependent on other users’ behaviour and would, therefore, bias the experimental comparisons made in the current paper. Current estimates provided by modern carbon trackers, including the one employed in this study, slightly underestimate power consumption for this reason. Our estimations did also not account for energy used for real-time interaction between software and hard disk storage. Prior research has shown this source of energy usage to be negligible in footprint estimations (~0.001 W per GB;Lannelongue et al., 2021). Future carbon tracking studies may also benefit from live tracking of energy usage over the course of a given computing job, providing access to timeseries data that would reveal how computational demands vary over time for a given piece of software. This was not possible with the carbon tracking tool used here. Third, while average file size was reported for both preprocessing and statistical analysis for each package, we did not report how file size generated varies between different specific preprocessing steps (e.g., realignment, registration, smoothing). While this was beyond the scope of the current paper, future research could benefit from incorporating this level of granularity. Finally, exact estimates of carbon emissions vary according to the carbon intensity of location and time^10^of computing, and power use effectiveness of the data centre. Our exact estimates are specific to computing conducted in the UK in 2024, with carbon intensity of 207.07, and on HPC architecture with PUE of 1.28. Estimates would be higher if computing was conducted in countries with higher carbon intensity (seeSouter et al., 2023) or in data centres with PUE closer to the industry standard of 1.55 (as of 2022; seeDavis et al., 2022). The exact size of the carbon footprint may also vary for those using personal computers rather than HPC infrastructure, as these computers are likely to use different processors. Personal computer users also do not need to factor in PUE, which will somewhat reduce total emissions. Carbon tracking tools such as Green Algorithms (https://calculator.green-algorithms.org/) allow users to observe differences in emissions as factors such as carbon intensity, PUE, and processor model vary. Despite these caveats, relative differences between packages observed in the current study should be reliable across different settings.

In conclusion, we have provided the first empirical comparison of estimated carbon emissions between fMRI software packages. fMRIPrep produced substantially higher emissions than FSL or SPM, and slightly higher statistical activation. Neuroimaging researchers can use these findings to inform their selection of fMRI data processing tools, considering their carbon footprint alongside ease of use, reproducibility, and the quality of the derived output. We encourage similar investigations of the carbon footprint associated with a wider range of neuroscience research tools, as well as of tools in other disciplines. To foster environmental sustainability, the neuroscience research community should support the development, optimisation, and use of tools that produce high-quality output while also minimising required compute.

## Ethics

No novel data were collected for this study, and as such it was not necessary to obtain ethical approval. All analysis was run using an existing open access dataset.

## Environmental Impact Statement

Across all participants and packages, preprocessing and statistical analysis of fMRI data for the current experiment used 25.06 kWh of energy over 2,296.52 hours, producing an estimated 6.38 kg of carbon dioxide equivalent emissions, determined using a server-side carbon tracker. Computing was conducted in the United Kingdom, with an average carbon intensity of 207.07 grams of CO_2_per kWh (http://www.carbonfootprint.com), on hardware with estimated power use effectiveness of 1.28.

## Supplementary Material

Supplementary Material

## Data Availability

Outcome data for each pipeline (preprocessing performance and carbon tracking metrics) are available on the Open Science Framework (OSF;https://osf.io/cdq6y). The data and code on this repository are sufficient to replicate statistical analysis of derived data and figure creation for this paper. Group-level fMRI maps are on Neurovault (https://neurovault.org/collections/QRJOSICN). Code used to run preprocessing and fMRI statistical analysis in each package and process the resulting data is available on GitHub (https://github.com/NickESouter/fMRI-Computing-Footprint).

## Footnotes

1 Assuming 108.1 gCO_2_/km, as taken from the 2022 average estimate of new passenger cars in Europe:https://www.eea.europa.eu/en/analysis/indicators/co2-performance-of-new-passenger

2
https://www.seagate.com/gb/en/esg/planet/product-sustainability/

3 Using the procedures outlined in the tutorial “*How to use FEAT while skipping registration*” from mumfordbrainstats;https://www.youtube.com/watch?v=U3tG7JMEf7M&t=482s

4 It is worth noting that the actual EVs provided in the public dataset linked toGorgolewski et al. (2017)do not conform to the design described in the paper. Each fixed duration is 1 second from the onset, accounting for the fixation cross and only half (500 ms) of the decision period. We have opted not to replicate this approach because (a) it would reduce statistical power by removing part of the trial from the model and (b) changing to the intended duration of 1.5 seconds should serve only to change the magnitude of the effect relative to the original dataset, rather than its qualitative nature (i.e., location of peak activation).

5 Despite carbon intensity typically falling each year in the UK in recent years, this is a 7% increase from the 2022 value (193.38) used in our previous study (Souter et al., 2024). As reported by the original source for this figure, this is attributable to “an increase in natural gas usage and decrease in renewables usage,” from 2022 to 2023 (Department for Energy Security & Net Zero, 2023, p.5).

6 t-statistics were extracted rather than z-statistics (as inSouter et al., 2024), given that statistical analysis in SPM produces t-statistic images but not z-statistic images.

7 Dice coefficients can range between 0 and 1 and reflect degree of overlap between maps, calculated using the formula: 2 x V_overlap_/(V1 + V2), where V_overlap_reflects size of overlap of two thresholded maps, and V1 and V2 reflect the size of the first and second maps, respectively. For further detail and examples of use, seeWilson et al. (2017)andBowring et al. (2019).

8
https://wellcome.org/grant-funding/guidance/policies-grant-conditions/environmental-sustainability-funding-policy

9
https://www.forbes.com/sites/alexanderpuutio/2024/12/22/ai-has-a-sustainability-problem-how-to-tackle-its-carbon-footprint/

10 To avoid systematic confounds, an average carbon intensity for the UK was used rather than live data (i.e.,https://carbonintensity.org.uk/)
