# Supplementary material for: Comparing the carbon footprint of fMRI data processing and analysis approaches
Source: Imaging Neurosci (Camb). 2025 Jun 16;3:IMAG.a.36. doi: 10.1162/IMAG.a.36 (PMC12319921; doi:10.1162/IMAG.a.36)
Supplement: Supplementary Material [file imag.a.36_supp.pdf]

Comparing the carbon footprint of fMRI data preprocessing and statistical analysis across software packages

Supplementary materials

Nicholas E. Souter [a], Chris Racey [a, b], Nikhil Bhagwat [c], Reese Wilkinson [d], Niall W. Duncan [e], Gabrielle Samuel [f], Loïc Lannelongue [g, h, i, j], Raghavendra Selvan [k], Charlotte L. Rae [a]

[a] School of Psychology, University of Sussex, Brighton, United Kingdom

[b] Sussex Neuroscience, University of Sussex, Brighton, United Kingdom

[c] McConnell Brain Imaging Centre, The Neuro (Montreal Neurological Institute - Hospital), McGill University; Montreal, Quebec, Canada

[d] Department of Physics and Astronomy, University of Sussex, Brighton BN1 9QH, UK

[e] Graduate Institute of Mind, Brain and Consciousness, Taipei Medical University, Taipei, Taiwan

[f] Department of Global Health and Social Medicine, King's College London, London, United Kingdom

[g] Cambridge Baker Systems Genomics Initiative, Department of Public Health and Primary Care, University of Cambridge, Cambridge, United Kingdom

[h] British Heart Foundation Cardiovascular Epidemiology Unit, Department of Public Health and Primary Care, University of Cambridge, Cambridge, United Kingdom

[i] Victor Phillip Dahdaleh Heart and Lung Research Institute, University of Cambridge, Cambridge, United Kingdom

[j] Health Data Research UK Cambridge, Wellcome Genome Campus and University of Cambridge, Cambridge, United Kingdom

[k] Department of Computer Science, University of Copenhagen, 2100, Copenhagen, Denmark

Deviations from preregistration

This project was preregistered on the OSF (https://osf.io/sqnbw) on January 19th, 2024. This preregistration was created using the Psychological Research Preregistration-Quantitative (PRP-QUANT) Template, version 2 (available at https://www.psycharchives.org). Several deviations were made from this preregistration:

(1) We had planned to include parametric regressors in first-level statistical analysis, relating to response time for ‘go’ and ‘unsuccessful stop’ trials, orthogonalised to the respective fixed duration regressor. We opted not to take this approach due to differences in handling or orthogonalisation in FSL FEAT and SPM (Mumford et al., 2015) which may unduly influence evidence of statistical activation. The original planned statistical analysis with parametric regressors is reported in supplementary section ‘*Analysis with parametric regressors*’.

(2) We had planned to use spherical ROIs when investigating statistical activation. These ROIs were created as part of Souter et al. (2024), and correspond to peaks of activation from the original statistical analysis of this data by Gorgolewski et al. (2017). We were concerned that this may bias evidence of activation towards data analysed in FSL FEAT (as in Gorgolewski et al., 2017). Instead, we therefore used regions from the Kong et al. (2021) 400-parcellation, corresponding anatomically to the regions we sought to probe.

(3) We had planned to analyse activation in the primary motor cortex and pre-SMA in the paper, leaving the insula and auditory cortex for the supplementary materials, as in Souter et al. (2024). On inspection of the data, it became clear that differences between packages varied in meaningful ways across regions. To ensure that package performance was compared as fairly and transparently as possible, all regions are instead analysed in the main paper. One omnibus ANOVA considering all regions together was performed instead of separate ANOVAs for each region, in order to look for an overall main effect of package across regions.

(4) As in Souter et al. (2024), we had planned to run a Bayesian repeated measures ANOVA for each frequentist ANOVA. These have now been removed for two reasons. First, while in Bayesian repeated measures ANOVAs, main effects (e.g., of package) can be easily interpreted, the interaction effects provided here are not truly analogous to those in frequentist models, meaning we would not be able to report Bayes factors for these

interactions. Second, the effect sizes observed in the frequentist ANOVAs are generally so large (often η_p^2 above .99), that Bayes factors are unlikely to provide additional insight into our confidence of these effects.

(5) We had planned to run all analysis in JASP, primarily because this provided a way of running Bayesian ANOVAs. Given that these Bayesian ANOVAs were removed, we opted to instead run all analysis directly in R, in order to reduce the number of files necessary to share to facilitate replication of our analysis. Non-parametric Wilcoxon comparisons were used for contrasts rather than t-tests given frequent violation of the normality assumption.

(6) We had planned to run fMRIPrep with the flag '--output-spaces MNI152NLin6Asym:res-2' corresponding to an output resolution of 2mm, interpreting this as the default setting. Given that the default is in fact native resolution in a different MNI space (MNI152NLin2009cAsym), we opted to use this latter setting to permit a fair comparison between packages. As a result, our expected sample size dropped from 257 to 248 as nine subjects' raw data did not have the same resolution as the remaining subjects.

(7) Finally, we had planned to use mean preprocessed data smoothness as an outcome measure alongside statistical task activation in the main manuscript. For reasons explained the Procedure section of the paper, analysis of this metric is now instead reported here in the Supplementary Materials, in the section '*Smoothness Estimation*'.

Supplementary Table 1. Flags passed to the command line for fMRIPrep

Flag	Description
--ignore **slicetiming**	• Tells fMRIPrep not to perform slicetiming correction – this step was not deemed necessary for the current study.
--track-carbon	• Runs CodeCarbon during preprocessing, providing a subject-specific estimate of total carbon emissions, duration, and energy consumption.
--country-code **GBR**	• Specifies the country in which CodeCarbon has been run using the relevant ISO Alpha-3 country code, in this case for Great Britain.
--random-seed **1234**	• Initiates a specific seed for the workflow. Along with the two following flags below, this helps to ensure reproducible preprocessing[1].
--skull-strip-fixed-seed	• Avoids using a random seed for skull-stripping. Again, this ensures reproducible preprocessing when used with the flag above and below.
--omp-nthreads **1**	• The maximum number of threads allocated per process. Setting this at a value of 1 ensures reproducible preprocessing when used with the two flags above.
--nthreads **5**	• The number of threads used across all processes, set at a default value of 5.
--mem-mb **3000**	• Sets an upper bound memory limit for fMRIPrep processes, at 3,000MB.
--skip-bids-validation	• Uses the assumption that input data is BIDS-valid (this was verified using the BIDS Validator prior to preprocessing; https://bids-standard.github.io/bids-validator/). When used, BIDS validation is skipped within fMRIPrep.
--fs-no-reconall	• Disables FreeSurfer surface reconstruction. Files generated by this step were not needed for the current study, and we have previously demonstrated that implementing this flag can reduce the emissions of fMRIPrep by 48% (Souter et al., 2024).

Note: This excludes positional arguments used to identify the location of relevant directories and licences, given that these are specific to the file paths and analysis servers we employed. Values or strings given to arguments (when relevant) are in bold. Carbon tracking in CodeCarbon was employed to allow for future comparison of emission estimated by this function and our own in-house tool. In practice, CodeCarbon estimates were not used in the current analysis.

[1] See https://mattermost.brainhack.org/brainhack/channels/fmriprep_reproducibility for a discussion on reproducibility within fMRIPrep.

fMRIPrep citation boilerplate

Results included in this manuscript come from preprocessing performed using fMRIPrep 22.1.1 (Esteban et al. (2019); Esteban et al. (2018); RRID:SCR_016216), which is based on Nipype 1.8.5 (K. Gorgolewski et al. (2011); K. J. Gorgolewski et al. (2018); RRID:SCR_002502).

Anatomical data preprocessing

A total of 1 T1-weighted (T1w) images were found within the input BIDS dataset. The T1-weighted (T1w) image was corrected for intensity non-uniformity (INU) with N4BiasFieldCorrection (Tustison et al. 2010), distributed with ANTs 2.3.3 (Avants et al. 2008, RRID:SCR_004757), and used as T1w-reference throughout the workflow. The T1w-reference was then skull-stripped with a Nipype implementation of the antsBrainExtraction.sh workflow (from ANTs), using OASIS30ANTs as target template. Brain tissue segmentation of cerebrospinal fluid (CSF), white-matter (WM) and gray-matter (GM) was performed on the brain-extracted T1w using fast (FSL 6.0.5.1:57b01774, RRID:SCR_002823, Zhang, Brady, and Smith 2001). Volume-based spatial normalization to one standard space (MNI152NLin2009cAsym) was performed through nonlinear registration with antsRegistration (ANTs 2.3.3), using brain-extracted versions of both T1w reference and the T1w template. The following template was selected for spatial normalization: ICBM 152 Nonlinear Asymmetrical template version 2009c [Fonov et al. (2009), RRID:SCR_008796; TemplateFlow ID: MNI152NLin2009cAsym].

Functional data preprocessing

For each of the 1 BOLD runs found per subject (across all tasks and sessions), the following preprocessing was performed. First, a reference volume and its skull-stripped version were generated using a custom methodology of fMRIPrep. Head-motion parameters with respect to the BOLD reference (transformation matrices, and six corresponding rotation and translation parameters) are estimated before any spatiotemporal filtering using mcflirt (FSL 6.0.5.1:57b01774, Jenkinson et al. 2002). The BOLD time-series (including slice-timing correction when applied) were resampled onto their original, native space by applying the transforms to correct for head-motion. These resampled BOLD time-series will be referred to as preprocessed BOLD in original space, or just preprocessed BOLD. The BOLD

reference was then co-registered to the T1w reference using mri_coreg (FreeSurfer) followed by flirt (FSL 6.0.5.1:57b01774, Jenkinson and Smith 2001) with the boundary-based registration (Greve and Fischl 2009) cost-function. Co-registration was configured with six degrees of freedom. Several confounding time-series were calculated based on the preprocessed BOLD: framewise displacement (FD), DVARS and three region-wise global signals. FD was computed using two formulations following Power (absolute sum of relative motions, Power et al. (2014)) and Jenkinson (relative root mean square displacement between affines, Jenkinson et al. (2002)). FD and DVARS are calculated for each functional run, both using their implementations in Nipype (following the definitions by Power et al. 2014). The three global signals are extracted within the CSF, the WM, and the whole-brain masks. Additionally, a set of physiological regressors were extracted to allow for component-based noise correction (CompCor, Behzadi et al. 2007). Principal components are estimated after high-pass filtering the preprocessed BOLD time-series (using a discrete cosine filter with 128s cut-off) for the two CompCor variants: temporal (tCompCor) and anatomical (aCompCor). tCompCor components are then calculated from the top 2% variable voxels within the brain mask. For aCompCor, three probabilistic masks (CSF, WM and combined CSF+WM) are generated in anatomical space. The implementation differs from that of Behzadi et al. in that instead of eroding the masks by 2 pixels on BOLD space, a mask of pixels that likely contain a volume fraction of GM is subtracted from the aCompCor masks. This mask is obtained by thresholding the corresponding partial volume map at 0.05, and it ensures components are not extracted from voxels containing a minimal fraction of GM. Finally, these masks are resampled into BOLD space and binarized by thresholding at 0.99 (as in the original implementation). Components are also calculated separately within the WM and CSF masks. For each CompCor decomposition, the k components with the largest singular values are retained, such that the retained components' time series are sufficient to explain 50 percent of variance across the nuisance mask (CSF, WM, combined, or temporal). The remaining components are dropped from consideration. The head-motion estimates calculated in the correction step were also placed within the corresponding confounds file. The confound time series derived from head motion estimates and global signals were expanded with the inclusion of temporal derivatives and quadratic terms for each (Satterthwaite et al. 2013). Frames that exceeded a threshold of 0.5 mm FD or 1.5 standardized DVARS were annotated as motion outliers. Additional nuisance timeseries are calculated by means of principal components analysis of the signal found within a thin band (crown) of voxels around the edge of the brain, as proposed by (Patriat, Reynolds, and Birn

2017). The BOLD time-series were resampled into standard space, generating a preprocessed BOLD run in MNI152NLin2009cAsym space. First, a reference volume and its skull-stripped version were generated using a custom methodology of fMRIPrep. All resamplings can be performed with a single interpolation step by composing all the pertinent transformations (i.e. head-motion transform matrices, susceptibility distortion correction when available, and co-registrations to anatomical and output spaces). Gridded (volumetric) resamplings were performed using antsApplyTransforms (ANTs), configured with Lanczos interpolation to minimize the smoothing effects of other kernels (Lanczos 1964). Non-gridded (surface) resamplings were performed using mri_vol2surf (FreeSurfer).

Many internal operations of fMRIPrep use Nilearn 0.9.1 (Abraham et al. 2014, RRID:SCR_001362), mostly within the functional processing workflow. For more details of the pipeline, see the section corresponding to workflows in fMRIPrep's documentation.

Copyright Waiver

The above boilerplate text was automatically generated by fMRIPrep with the express intention that users should copy and paste this text into their manuscripts unchanged. It is released under the CC0 license.

Effects of fMRIPrep output space resolution

In previous work (Souter et al., 2024), we carbon tracked fMRIPrep outputting to a standard 2mm output space. By default, fMRIPrep in fact outputs data in subjects' native resolution (in this case, 3x3x4mm) - this was the approach taken in the current paper to allow for a fair comparison of default configurations between packages. To quantify the effect of this preprocessing choice, here we present a comparison of carbon tracking and task performance metrics for fMRIPrep in native resolution and 2mm resolution, both run on the same 248 subjects referenced in the paper, with all other fMRIPrep settings kept as default. All dependent variables derived were extracted using the same procedure detailed for fMRIPrep data in the main paper.

As well as increasing output resolution, the 2mm version uses the 'MNI152NLin6Asym' output space (formatted in the command line as '--output-spaces MNI152NLin6Asym:res-2') rather than the default 'MNI152NLin2009cAsym' provided by fMRIPrep. We do not anticipate this is likely to confound comparison of these pipelines.

In Supplementary Figure 1, we present bar plots reflecting each dependent variable for fMRIPrep in both native and 2mm resolution, alongside data for FSL and SPM. In Supplementary Table 2, we present repeated measures ANOVAs used to observe effects of package on each dependent variable, restricted to comparison on fMRIPrep and fMRIPrep2mm (excluding FSL and SPM). When possible, we tested main effects and interactions of stage, energy source, and region. All dependent variables were significantly influenced by each of the relevant independent variables.

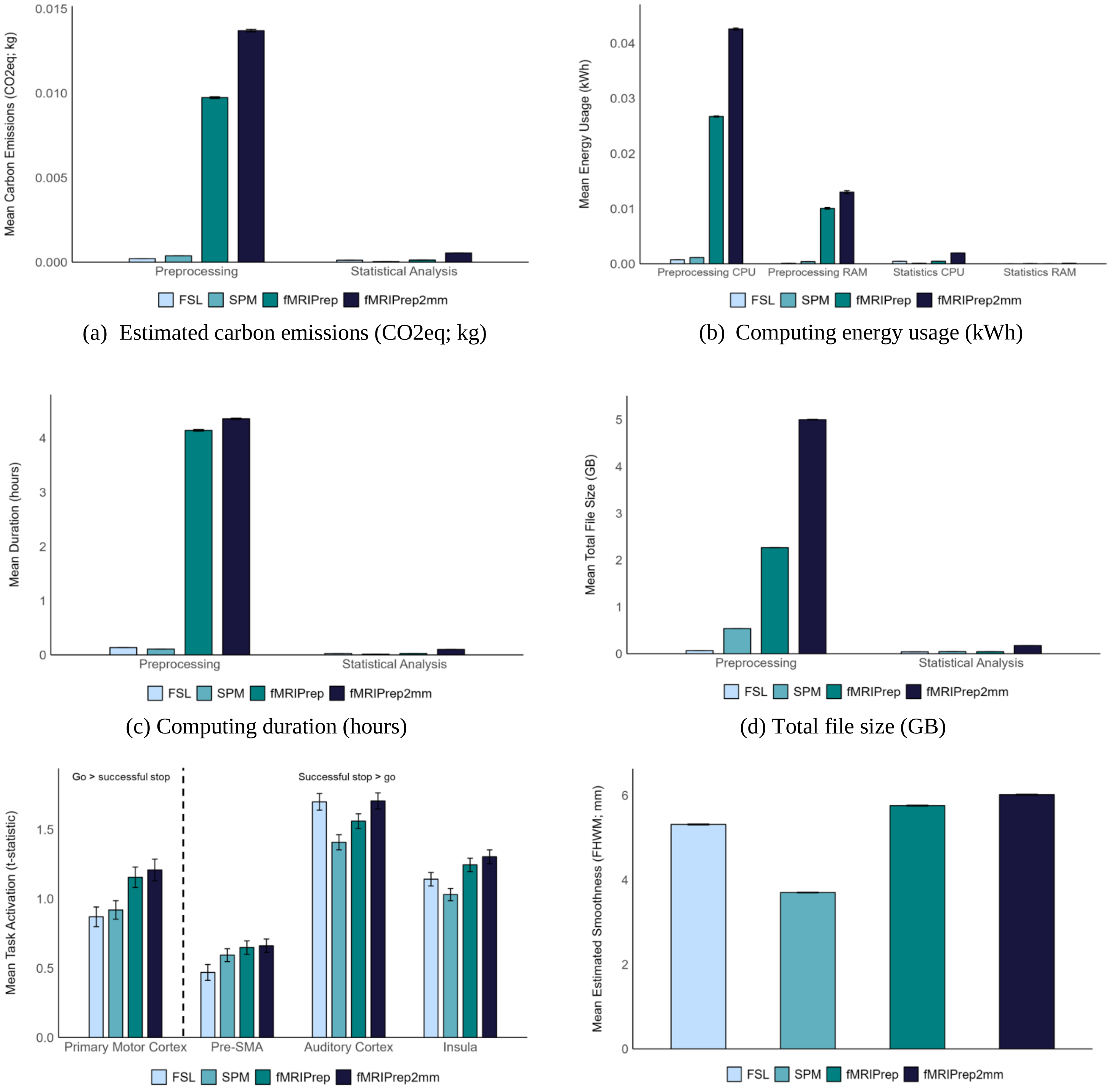

(a) Estimated carbon emissions (CO2eq; kg)

(b) Computing energy usage (kWh)

(c) Computing duration (hours)

(d) Total file size (GB)

(e) Statistical task activation (t-statistic)

(f) Estimated smoothness (FWHM; mm)

Supplementary Figure 1. Mean values (per participant) for each package, including fMRIPrep with data outputted to a standard 2mm output space (fMRIPrep2mm) rather than default native resolution (3x3x4mm). Data presented for (a) estimated carbon emissions, (b) energy usage, (c) duration of computing, (d) total file size, (e) statistical activation in regions of interest (regions split according to the contrast they were interrogated with respect to), and (f) mean data smoothness. Metrics split by stage (preprocessing/statistical analysis), energy source (CPU/RAM), and region of interest when appropriate. Error bars reflect one standard error of the mean. These are frequently too small to be visible. Pre-SMA = pre-supplementary motor area, CPU = central processing unit, RAM = random-access memory, CO2eq = carbon dioxide equivalent, mm = millimetres, kWh = kilowatt hours, kg = kilograms; FWHM = full width half maximum

Supplementary Table 2. Repeated measures ANOVAs for carbon emissions, energy usage, duration, total file size, task activation, and smoothness for fMRIPrep in native and 2mm resolution

Variable	Effect	Result
Carbon emissions	fMRIPrep version	$F(1, 234) = 2{,}220.5$, $p < .001$*, $\eta_p^2 = .90$
	Stage	$F(1, 234) = 62{,}345.8$, $p < .001$*, $\eta_p^2 = .996$
	fMRIPrep version x stage	$F(1, 234) = 1{,}452.6$, $p < .001$*, $\eta_p^2 = .86$
Energy usage	fMRIPrep version	$F(1, 223) = 3{,}085.0$, $p < .001$*, $\eta_p^2 = .93$
	Stage	$F(1, 223) = 63{,}300.3$, $p < .001$*, $\eta_p^2 = .996$
	Source	$F(1, 223) = 25{,}060.0$, $p < .001$*, $\eta_p^2 = .99$
	fMRIPrep version x stage	$F(1, 223) = 2{,}204.2$, $p < .001$*, $\eta_p^2 = .91$
	fMRIPrep version x source	$F(1, 223) = 1{,}850.3$, $p < .001$*, $\eta_p^2 = .89$
	Stage x source	$F(1, 223) = 20{,}657.3$, $p < .001$*, $\eta_p^2 = .99$
	fMRIPrep version x stage x source	$F(1, 223) = 1{,}165.8$, $p < .001$*, $\eta_p^2 = .84$
Duration	fMRIPrep version	$F(1, 235) = 255.4$, $p < .001$*, $\eta_p^2 = .52$
	Stage	$F(1, 235) = 164{,}402.5$, $p < .001$*, $\eta_p^2 = .999$
	fMRIPrep version x stage	$F(1, 235) = 61.5$, $p < .001$*, $\eta_p^2 = .21$
Total file size	fMRIPrep version	$F(1, 240) = 158{,}866.2$, $p < .001$*, $\eta_p^2 = .998$
	Stage	$F(1, 240) = 662{,}608.3$, $p < .001$*, $\eta_p^2 > .999$
	fMRIPrep version x stage	$F(1, 240) = 129{,}973.5$, $p < .001$*, $\eta_p^2 = .998$
Task activation	fMRIPrep version	$F(1, 244) = 198.5$, $p < .001$*, $\eta_p^2 = .45$
	Region [a]	$F(1.9, 466.1) = 49.4$, $p < .001$*, $\eta_p^2 = .17$
	fMRIPrep version x Region [a]	$F(2.4, 595.1) = 32.8$, $p < .001$*, $\eta_p^2 = .12$
Smoothness	Package	$F(1, 246) = 10{,}959.3$, $p < .001$*, $\eta_p^2 = .98$

Notes: * reflects significant results at $p < .05$. [a] reflects Greenhouse-Geisser correction applied due to violation of the assumption of sphericity. Levels for effects are as follows: fMRIPrep version (Native, 2mm), Stage (Preprocessing, Statistical analysis), Source (CPU, RAM), Region (Primary motor cortex, Pre-supplementary motor area, Auditory cortex, Insula). Results for Carbon emissions and Energy usage are comparable given that values for these variables are exactly proportional.

Effects and interactions for each dependent variable are parsed below. In short, requesting 2mm output resolution increased estimated carbon emissions by 40% and more than doubled total file size. At the same time, increasing resolution modestly improved statistical sensitivity in regions of interest, by an average of 5.5%. From our experience, outputting to 2mm resolution may provide ease of use benefits, such as easier interfacing and analysis with existing region of interest files that are released in 2mm. However, users should be aware that doing so comes with computational costs.

We parse each interaction which contains ‘fMRIPrep version’ as a term, given that comparisons between the performance of fMRIPrep in native and 2mm resolution were of interest here. For simplicity, these two variants of fMRIPrep are referred to as ‘Native’ and ‘2mm’, respectively. The descriptive relative difference between the relevant levels of a given variable are reported in each case, as well as non-parametric Wilcoxon contrasts (when needed) with false discovery rate (FDR) correction using the Benjamini-Hochberg method applied for contrasts within a given effect/interaction.

Estimated carbon emissions for 2mm were 1.4x higher than for Native. Emissions were higher for preprocessing than for statistical analysis, with this difference being larger for Native (76x; Z = -13.57, $p < .001$) than for 2mm (25x; Z = -13.37, $p < .001$).

Energy usage for 2mm was 1.5x greater than for Native. Energy usage was greater for preprocessing than statistical analysis, with this difference larger for Native (76x; Z = -13.26, $p < .001$) than for 2mm (27x; Z = -13.37, $p < .001$). Energy usage was greater for CPU than for RAM, with this difference greater for 2mm (3.4x; Z = -13.37, p < .001) than for Native (2.7x; Z = -13.26, $p < .001$). As demonstrated by an interaction of fMRIPrep version, stage, and source, energy usage was greater for CPU than RAM for each combination of fMRIPrep version and stage, with the extent of this difference varying by fMRIPrep version and stage; Native preprocessing (2.7x; Z = -13.60, $p < .001$), Native stats (23x; Z = -13.32, $p < .001$), 2mm preprocessing (3.3x; Z = -13.51, $p < .001$), 2mm stats (17x; Z = -13.51, $p < .001$).

Duration of computing for 2mm was 1.1x longer than for Native. Duration was longer for preprocessing than for statistical analysis, with this difference being larger for Native (150x; Z = -13.57, $p < .001$) than for 2mm (43x; Z = -13.40, $p < .001$).

File size for 2mm was 2.2x greater than for Native. File size was larger for preprocessing than for statistical analysis, with this difference being greater for Native (53x; Z = -13.49 , $p < .001$) than for 2mm (29x; Z = -13.57, $p < .001$).

Overall task activation across regions was 1.1x greater (specifically, +6%) for 2mm than for Native. This direction of effect held true for three regions, but the magnitude of difference varied: Primary motor cortex (1.05x; Z = -5.19, $p < .001$), Auditory cortex (1.1x; Z = -12.53, $p < .001$), Insula (1.05x; Z = -8.56, $p < .001$). No significant difference between fMRIPrep versions was observed for the pre-SMA (Z = -1.14, $p = .255$).

Mean data smoothness for 2mm was 1.05x higher than for Native.

Analysis with parametric regressors

When preregistering this study, we had planned to include parametric modulations relating to response time (RT) in the fMRI analysis model design for data processed in each package. Due to differences in the handling of parametric modulation and orthogonalisation across packages (Mumford et al., 2015), we ultimately excluded these parametric regressors from the model. Here, we present analysis equivalent to our assessment of mean t-statistic in regions of interest (ROIs) in the paper (our main quantitative measure of performance) but with parametric modulators included in the model. The model design used in this version of the analysis was the same as that used for this same data by Gorgolewski et al. (2017) and Esteban et al. (2019). Six regressors were created:

1. Go – Trials in the 'go' condition for which participants responded correctly, with a fixed duration of 1.5 seconds.
2. Go RT – Trials in the 'go' condition for which participants responded correctly. Duration was set as the subject's response time (RT) for the respective trial.
3. Successful stop – Trials in the 'stop' condition for which participants successfully inhibited their motor response, with a fixed duration of 1.5 seconds.
4. Unsuccessful stop – Trials in the 'stop' condition for which participants did not successfully inhibit their motor response, with a fixed duration of 1.5 seconds.
5. Unsuccessful stop RT – Trials in the 'stop' condition for which participants did not successfully inhibit their motor response. Duration was set as the subject's RT for the respective trial.
6. Erroneous – Trials in the 'go' condition for which participants responded incorrectly, with a fixed duration of 1.5 seconds. Ninety-nine participants had no erroneous 'go' trials. In these cases, task data was modelled with 5 EVs, with 'erroneous' omitted.

The onset time of each regressor was set as the start of the 0.5 second fixation that preceded each decision period[2]. Time periods not covered by the EVs include null periods

[2] It is worth noting that the actual EVs provided in the public dataset linked to Gorgolewski et al. (2017) do not conform to the design described in the paper. Each fixed duration is 1 second from the onset, accounting for the fixation cross and only half (500ms) of the decision period. We have opted not to replicate this approach because (a) it would reduce statistical power by removing part of the trial from the model and (b) changing to the intended duration of 1.5 seconds should serve only to change the magnitude of the effect relative to the original dataset, rather than its qualitative nature (i.e., location of peak activation).

between trials, and ‘go’ trials on which participants provided no response (only 76 participants had any instance of this).

‘Go RT’ and ‘Unsuccessful stop RT’ were both orthogonalised to the respective fixed duration regressor of the same condition. In SPM, this involved setting RT duration as a ‘Parametric Modulation’ for the respective condition, with ‘Orthogonalise modulations’ set to ‘Yes’. In FSL FEAT (covering data preprocessed in both FSL and fMRIPrep), RT regressors were entered as separate EVs in the model, and set as orthogonalised to the respective condition in the GUI.

As in Esteban et al. (2019), we extracted the contrast of ‘go > successful stop’, producing a statistical image in which positive values reflected voxels activated by motor response, and negative values reflected voxels activated by successful response inhibition. The reverse of this contrast was also extracted to provide ‘successful stop > go’. We then extracted mean statistical activation in task-relevant ROIs, using the same procedure and regions detailed in the main paper (see section 4.4.6).

Supplementary Figure 2 provides a bar plot of mean statistical activation for each ROI in each package with the inclusion of parametric modulations in the model design. To aid comparison of this data to equivalent values without parametric modulation included in the model (see Figure 3e), Supplementary Table 3 presents descriptives statistics for the mean t-statistic in each ROI for each package, with and without parametric modulations, as well as the relative difference (% change).

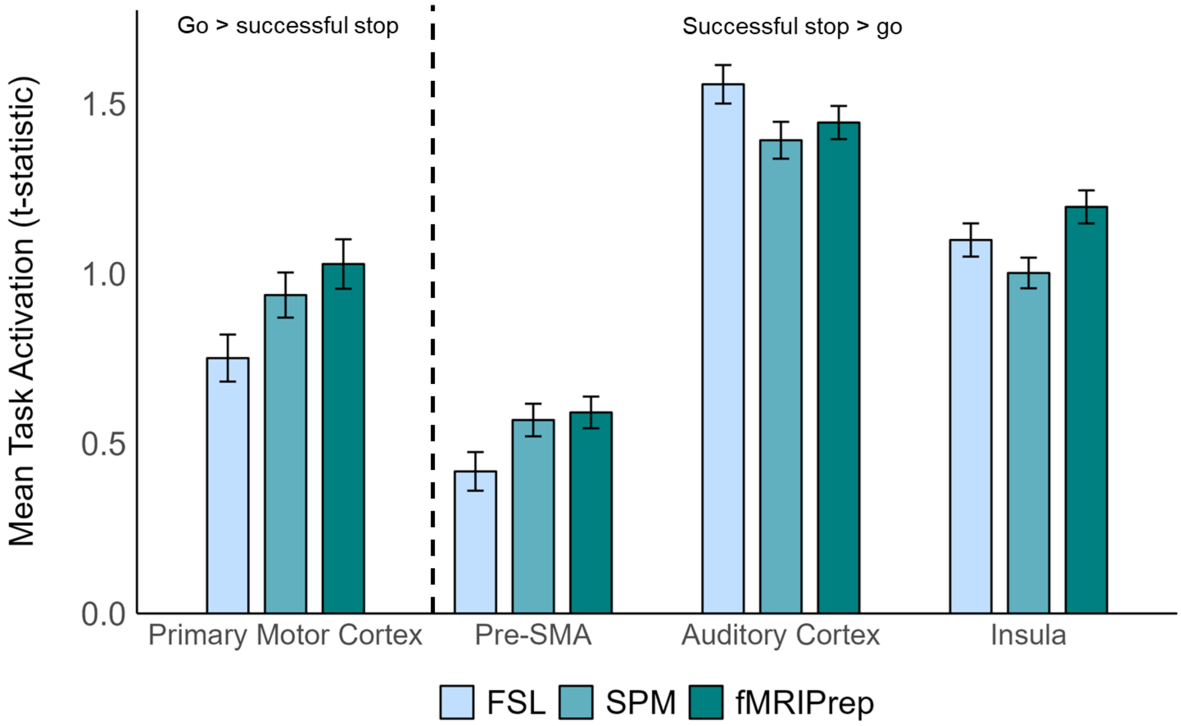

Supplementary Figure 2. Mean task activation (t-statistic) in regions of interest for each package with the inclusion of response time as a parametric modulation. Regions are split according to whether they were interrogated with respect to the 'go > successful stop' or 'successful stop > go' contrast. Error bars reflect one standard error of the mean. SMA = supplementary motor area

Supplementary Table 3. Mean t-statistics without and with the inclusion of response time parametric modulation in the model design and relative percent change

		Mean t-statistic		
Package	Region	No parametric modulation	Parametric modulation	% change
FSL	Primary motor cortex	0.87	0.75	-13.70%
	Pre-SMA	0.47	0.42	-10.95%
	Auditory cortex	1.70	1.56	-8.39%
	Insula	1.14	1.10	-3.77%
SPM	Primary motor cortex	0.92	0.94	+1.83%
	Pre-SMA	0.59	0.57	-4.22%
	Auditory cortex	1.41	1.39	-1.13%
	Insula	1.03	1.00	-2.83%
fMRIPrep	Primary motor cortex	1.16	1.03	-11.05%
	Pre-SMA	0.65	0.59	-8.85%
	Auditory cortex	1.56	1.44	-7.46%
	Insula	1.24	1.20	-3.94%

Note: % change reflects the relative change in mean t-statistic when parametric modulation is included. Cases of relative decrease (poorer performance) are highlighted in red and relative increase (improved performance) in blue. Pre-SMA = pre-supplementary motor area.

Supplementary Table 4 provides analysis (using frequentist repeated measures ANOVA) of the effect of modulation on mean statistical activation, by comparing this data to that without the inclusion of parametric modulation (see Figure 3e).

Supplementary Table 4. Repeated measures ANOVAs observing effects and interactions of package, region, and parametric modulation on mean statistical activation in regions of interest

Effect	Result
Package [a]	F(1.9, 443.8) = 31.7, $p < .001$*, $\eta_p^2 = .12$
Region [a]	F(2.0, 475.0) = 57.2, $p < .001$*, $\eta_p^2 = .19$
Modulation	F(1, 239) = 65.1, $p < .001$*, $\eta_p^2 = .21$
Package x region [a]	F(4.8, 1,137.1) = 21.8, $p < .001$*, $\eta_p^2 = .08$
Package x modulation [a]	F(1.3, 318.7) = 40.9, $p < .001$*, $\eta_p^2 = .15$
Region x modulation [a]	F(1.7, 406.6) = 3.2, $p = .050$, $\eta_p^2 = .01$
Package x region x modulation [a]	F(2.4, 570.0) = 7.8, $p < .001$*, $\eta_p^2 = .03$

Note: * reflects significant results at $p < .05$. [a] Greenhouse-Geisser correction applied due to violation of the assumption of sphericity. Levels for effects are as follows: Package (FSL, SPM, fMRIPrep), Region (Primary motor cortex, Pre-supplementary motor area, Auditory cortex, Insula), Modulation (With, Without).

While most effects and interactions were found to be significant, of interest here was the relative effect of parametric modulation on each package. As such, contrasts (using FDR correction with the Benjamini-Hochberg method) were only used to parse the interaction of package and modulation. This revealed significant decreases in statistical sensitivity following the introduction of parametric modulation for all packages (FSL: Z = -6.91, $p < .001$, SPM: Z = -2.48, $p = .013$, fMRIPrep: Z = -7.34, $p < .001$). The average difference between values for analysis with and without parametric modulation was largest for FSL (.045), followed by fMRIPrep (.044), and then SPM (.007).

Therefore, for this data, negative effects of including RT as a parametric modulation are greater for data statistically analysed in FSL FEAT than for data analysed in SPM. This may be due to differences in the ways in which FSL and SPM handle parametric regressors and orthogonalisation (Mumford et al., 2015). Unless necessary in model design (e.g., investigating parametric effects of RT on task activation), neuroimagers should reflect on whether inclusion of such parametric modulations is appropriate in their own models.

Effect of fMRIPrep ROI transformation

Supplementary Table 5. Effect of ANTs transformation on regions of interest for Featquery analysis of fMRIPrep data

Region	Standard resolution (2x2x2mm)		Native resolution (3x3x4mm)		Volume percent change
	Voxels	Volume (mm^3)	Voxels	Volume (mm^3)	
Left primary motor cortex	30	1,080	125	1,000	-7.69%
Right Pre-supplementary motor area	39	1,404	205	1,640	+15.51%
Left auditory cortex	130	4,680	490	3,920	-17.67%
Right insula	102	3,672	430	3,440	-6.52%

Note: Regions were transformed from standard to native resolution and to the MNI152NLin2009cAsym output space. Values provided are the size of regions of interest in voxels, and the volume im mm3 of each region (number of voxels multiplied by cubed resolution). Volume percent change reflects the percentage change in volume as a result of ANTs transformation.

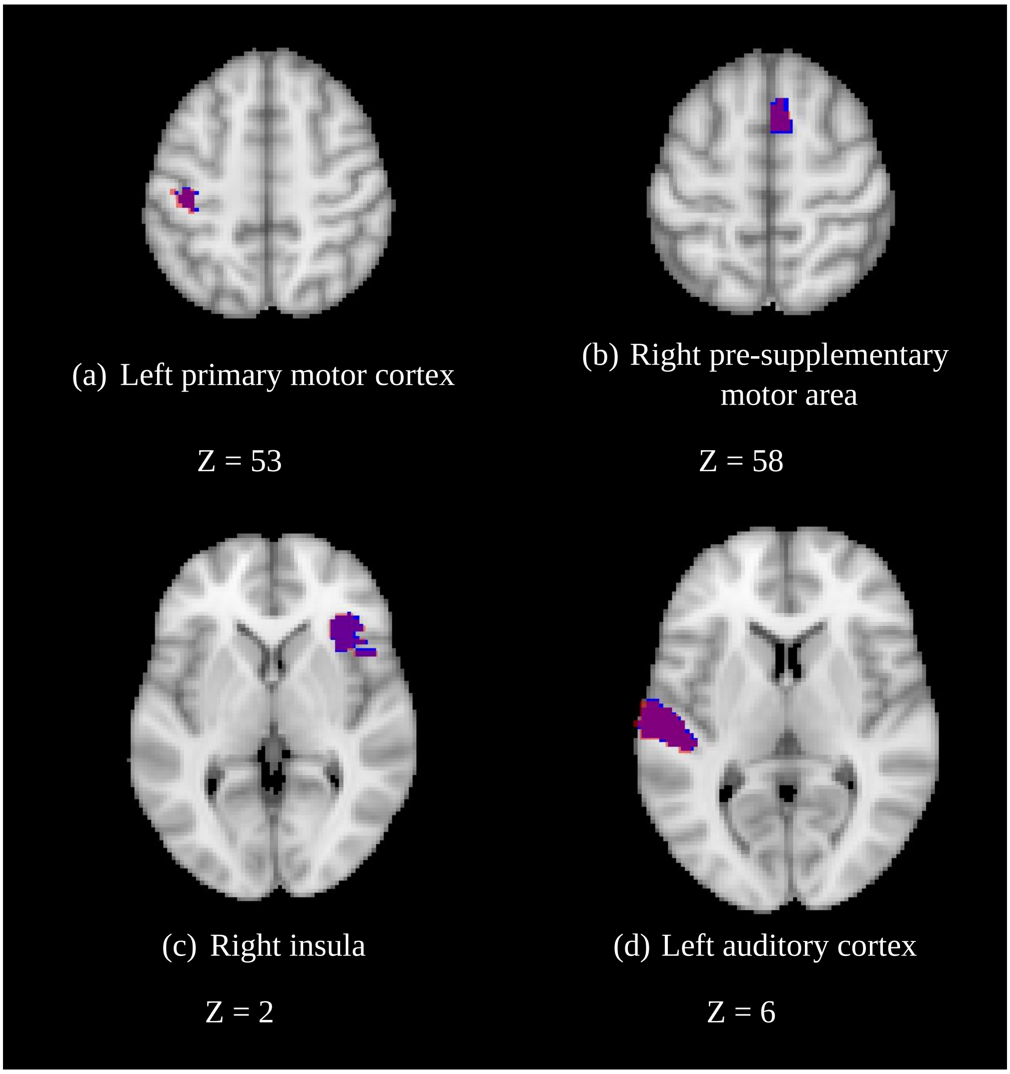

Supplementary Figure 3. Effects of ANTs transformation of regions of interest, for use in fMRIPrep. Transformations to native resolution (red) are transparent and overlayed over original standard resolution regions (blue) in FSLeyes. While FSLeyes allows visual comparisons of these regions, note that it was not possible to produce this figure in R as has been done for other figures in this paper due to the difference in the underlying resolution of the NIFTI files.

Smoothness Estimation

As a second measure of preprocessing performance (alongside statistical task activation), the mean smoothness of data preprocessed in each package was estimated in AFNI. Mean geometric smoothness estimates were extracted for each participant's preprocessed and smoothed fMRI data, using AFNI's 3dFWHMx. Outlier voxels were accounted for using the 'detrend' flag. This tool provides a mean smoothness estimate for each of the 184 volumes of the fMRI run on the x, y, and z dimension. For each dimension, outlier smoothness estimates +/- three standard deviations from the dimension mean were removed. Values were then averaged within and across dimensions to produce a single mean smoothness estimate. As in Esteban et al. (2019), larger mean smoothness values were taken as reflecting poorer pipeline performance, on the basis that increased smoothness reflects loss of anatomical specificity and statistical power. In Souter et al. (2024), we extracted estimates of smoothness for both pre- and post-smoothed data. In the current study, this was not possible for data preprocessed and smoothed in FSL FEAT, given a lack of an unsmoothed preprocessed file. As such, only post-smoothed smoothness is reported and compared here.

As for other dependent variables in the manuscript, we observed the effect of software package (FSL, SPM, fMRIPrep) on mean smoothness, using repeated measures ANOVA, run in R (version 4.2.2) using the rstatix package (version 0.7.2; https://rpkgs.datanovia.com/rstatix). Prior to analysis, outliers (greater than three standard deviations above or below the group mean) for each level of the respective variable for the respective package were removed.

Supplementary Figure 4 presents mean smoothness values (per participant) for each package.

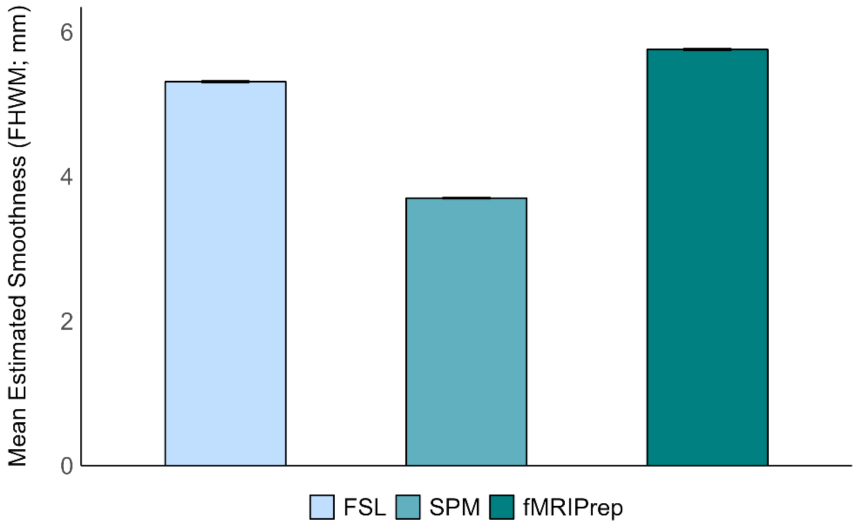

Supplementary Figure 4. Mean estimated smoothness values (per participant) for each package. Error bars reflect one standard error of the mean; these are too small to be visible. mm = millimetres, FWHM = full width half maximum

Smoothness was significantly influenced by package; F(1.3, 316.3) = 26,852.4, $p < .001$*, $\eta_p^2 = .99$. Note that Greenhouse-Geisser correction was applied due to violation of the assumption of sphericity. At the level of specific package comparisons, mean smoothness was higher for fMRIPrep than FSL (1.1x) and SPM (1.6x), and for FSL than SPM (1.4x). We took greater smoothness to reflect poorer spatial resolution, and therefore poorer performance. However, performance according to smoothness appears inverse to task activation (see Figure 3). Improved signal to noise ratio as a result of smoothness may outweigh the downsides of decreased spatial resolution (see Caballero-Gaudes & Reynolds, 2017; Triantafyllou et al., 2006). Note that statistical comparison of smoothness estimates may be influenced by differences in the underlying resolution of the data for each package. Supplementary Figure 5 presents a histogram of mean smoothness values for each package, for a more descriptive comparison of values.

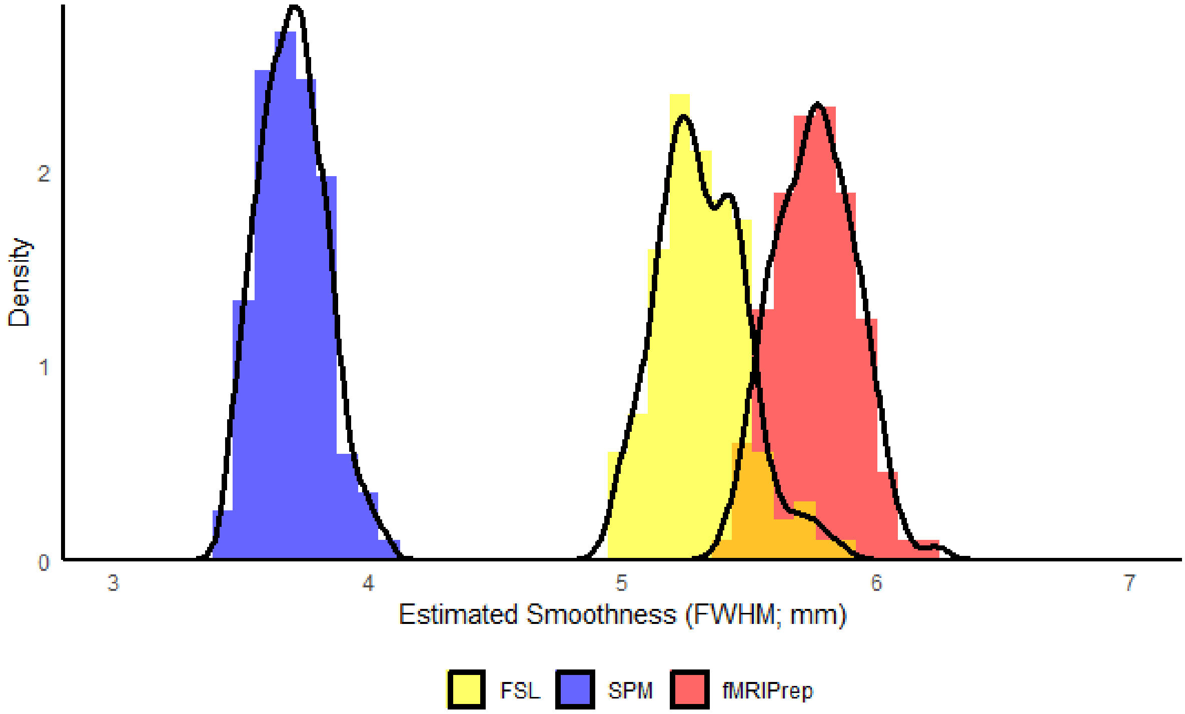

Supplementary Figure 5. Histogram of mean smoothness estimates for smoothed data for each software package, comparable to that presented by Esteban et al. (2019). FWHM = full-width half maximum

Supplementary Table 6. Planned contrasts for significant frequentist ANOVA, for each dependent variable

Variable	Effect	Comparison	Result
Carbon emissions	Package	**FSL vs SPM**	**Z = -13.32, *p* < .001***
		**FSL vs fMRIPrep**	**Z = -13.35, *p* < .001***
		**SPM vs fMRIPrep**	**Z = -13.49, *p* < .001***
	Package x stage	**FSL: Preprocessing vs Statistical analysis**	**Z = -13.43, *p* < .001***
		**SPM: Preprocessing vs Statistical analysis**	**Z = -13.57, *p* < .001***
		**fMRIPrep: Preprocessing vs Statistical analysis**	**Z = -13.57, *p* < .001***
Energy usage	Package	**FSL vs SPM**	**Z = -13.30, *p* < .001***
		**FSL vs fMRIPrep**	**Z = -13.06, *p* < .001***
		**SPM vs fMRIPrep**	**Z = -13.15, *p* < .001***
	Package x stage	**FSL: Preprocessing vs Statistical analysis**	**Z = -13.43, *p* < .001***
		**SPM: Preprocessing vs Statistical analysis**	**Z = -13.54, *p* < .001***
		**fMRIPrep: Preprocessing vs Statistical analysis**	**Z = -13.26, *p* < .001***
	Package x source	**FSL: CPU vs RAM**	**Z = -13.43, *p* < .001***
		**SPM: CPU vs RAM**	**Z = -13.54, *p* < .001***
		**fMRIPrep: CPU vs RAM**	**Z = -13.26, *p* < .001***
	Stage x source	**Preprocessing: CPU vs RAM**	**Z = -13.49, *p* < .001***
		**Statistical analysis: CPU vs RAM**	**Z = -13.09, *p* < .001***
	Package x stage x source	**FSL: Preprocessing: CPU vs RAM**	**Z = -13.60, *p* < .001***
		**FSL: Statistical analysis: CPU vs RAM**	**Z = -13.49, *p* < .001***
		**SPM: Preprocessing: CPU vs RAM**	**Z = -13.60, *p* < .001***
		**SPM: Statistical analysis: CPU vs RAM**	**Z = -13.60, *p* < .001***
		**fMRIPrep: Preprocessing: CPU vs RAM**	**Z = -13.60, *p* < .001***
		**fMRIPrep: Statistical analysis: CPU vs RAM**	**Z = -13.32, *p* < .001***
Duration	Package	**FSL vs SPM**	**Z = -13.40, *p* < .001***
		**FSL vs fMRIPrep**	**Z = -13.37, *p* < .001***
		**SPM vs fMRIPrep**	**Z = -13.51, *p* < .001***
	Package x stage	**FSL: Preprocessing vs Statistical analysis**	**Z = -13.46, *p* < .001***
		**SPM: Preprocessing vs Statistical analysis**	**Z = -13.60, *p* < .001***
		**fMRIPrep: Preprocessing vs Statistical analysis**	**Z = -13.57, *p* < .001***
File size	Package	**FSL vs SPM**	**Z = -13.62, *p* < .001***
		**FSL vs fMRIPrep**	**Z = -13.46, *p* < .001***
		**SPM vs fMRIPrep**	**Z = -13.46, *p* < .001***
	Package x stage	**FSL: Preprocessing vs Statistical analysis**	**Z = -13.62, *p* < .001***
		**SPM: Preprocessing vs Statistical analysis**	**Z = -13.62, *p* < .001***
		**fMRIPrep: Preprocessing vs Statistical analysis**	**Z = -13.49, *p* < .001***
Task activation	Package	**FSL vs SPM**	**Z = -3.30, *p* < .001***
		**FSL vs fMRIPrep**	**Z = -5.37, *p* < .001***
		**SPM vs fMRIPrep**	**Z = -9.69, *p* < .001***
	Package x region	Primary motor cortex: FSL vs SPM	Z = -1.32, *p* = .186
		**Primary motor cortex: FSL vs fMRIPrep**	**Z = -6.54, *p* < .001***
		**Primary motor cortex: SPM vs fMRIPrep**	**Z = -6.74, *p* < .001***
		**Pre-SMA: FSL vs SPM**	**Z = -3.04, *p* = .003***
		**Pre-SMA: FSL vs fMRIPrep**	**Z = -4.15, *p* < .001***
		**Pre-SMA: SPM vs fMRIPrep**	**Z = -2.62, *p* = .009***
		**Auditory cortex: FSL vs SPM**	**Z = -8.40, *p* < .001***
		**Auditory cortex: FSL vs fMRIPrep**	**Z = -5.34, *p* < .001***
		**Auditory cortex: SPM vs fMRIPrep**	**Z = -6.60, *p* < .001***
		**Insula: FSL vs SPM**	**Z = -3.11, *p* = .003***
		**Insula: FSL vs fMRIPrep**	**Z = -3.52, *p* < .001***
		**Insula: SPM vs fMRIPrep**	**Z = -7.24, *p* < .001***

Smoothness	Package	**FSL vs SPM**	**Z = -13.57, *p* < .001***
		**FSL vs fMRIPrep**	**Z = -13.57, *p* < .001***
		**SPM vs fMRIPrep**	**Z = -13.62, *p* < .001***

Note: * reflects a significant result at $p < .05$. Significant results are also in bold. For significant effects, levels for which the respective value is higher is highlighted in blue. Contrasts are corrected for multiple comparisons using False Discovery Rate correction using the Benjamini-Hochberg method, within each effect for each variable. CPU = central processing unit, RAM = random access memory.

Group-Level quantitative comparison

Supplementary Table 7. Computing metrics for group-level statistical analysis in each package

	Estimated Carbon emissions (kg)	CPU energy usage (kWh)	RAM energy usage (kWh)	Duration (hours)	File size (GB)
FSL	.02640	.09413	.00547	4.90	4.68
SPM	.00007	.00015	.00013	0.03	0.04
fMRIPrep [a]	.00498	.01778	.00100	1.34	0.84

Note: [a] first- and group-level analysis for fMRIPrep was conducted in FSL FEAT, given that fMRIPrep does not include a statistical analysis component. For data statistically analysed in FSL FEAT (FSL and fMRIPrep), file size includes individual-level registrations to standard space, which are generated during group-level statistical analysis.

Supplementary Table 8. Size and overlap extent (dice coefficients) of thresholded group-level activation maps

Contrast	Package	Size (voxels)	Dice coefficient			% outside brain mask
			FSL	SPM	fMRIPrep	
Go > Successful stop	FSL	22,067	-	0.47	0.52	10.45%
	SPM	22,929	0.47	-	0.51	1.09%
	fMRIPrep	15,748	0.52	0.51	-	1.09%
Successful stop > Go	FSL	81,778	-	0.60	0.72	6.41%
	SPM	52,357	0.60	-	0.63	0.22%
	fMRIPrep	58,671	0.72	0.63	-	0.71%

Note: Dice coefficients can range between 0 and 1 and reflect degree of overlap between maps, calculated using the formula: 2 x $V_{overlap}$ /(V1 + V2), where $V_{overlap}$ reflects size of overlap of two thresholded maps, and V1 and V2 reflect the size of the first and second maps, respectively. % outside brain mask reflects the percentage of thresholded voxels for a given map that fall outside of the MNI 2mm brain template provided by FSL.

References

Abraham, A., Pedregosa, F., Eickenberg, M., Gervais, P., Mueller, A., Kossaifi, J., Gramfort, A., Thirion, B., & Varoquaux, G. (2014). Machine learning for neuroimaging with scikit-learn. *Frontiers in Neuroinformatics*, *8*. https://doi.org/10.3389/fninf.2014.00014

Avants, B. B., Epstein, C. L., Grossman, M., & Gee, J. C. (2008). Symmetric diffeomorphic image registration with cross-correlation: Evaluating automated labeling of elderly and neurodegenerative brain. *Medical Image Analysis*, *12*(1), 26–41. https://doi.org/10.1016/j.media.2007.06.004

Behzadi, Y., Restom, K., Liau, J., & Liu, T. T. (2007). A component based noise correction method (CompCor) for BOLD and perfusion based fMRI. *NeuroImage*, *37*(1), 90–101. https://doi.org/10.1016/j.neuroimage.2007.04.042

Esteban, O., Blair, R., Markiewicz, C. J., Berleant, S. L., Moodie, C., Ma, F., Isik, A. I., et al. (2018). fMRIPrep 22.1.1 [*Software*]. https://doi.org/10.5281/zenodo.852659

Esteban, O., Markiewicz, C. J., Blair, R. W., Moodie, C. A., Isik, A. I., Erramuzpe, A., Kent, J. D., Goncalves, M., DuPre, E., Snyder, M., Oya, H., Ghosh, S. S., Wright, J., Durnez, J., Poldrack, R. A., & Gorgolewski, K. J. (2019). fMRIPrep: a robust preprocessing pipeline for functional MRI. *Nature Methods*, *16*, 111-116. https://doi.org/10.1038/s41592-018-0235-4

Fonov, V. S., Evans, A. C., McKinstry, R. C., Almli, C. R., & Collins, D. L. (2009). Unbiased nonlinear average age-appropriate brain templates from birth to adulthood. *NeuroImage*, *47*(Suppl 1), S102. https://doi.org/10.1016/S1053-8119(09)70884-5

Gorgolewski, K., Burns, C. D., Madison, C., Clark, D., Halchenko, Y. O., Waskom, M. L., & Ghosh, S. (2011). Nipype: A flexible, lightweight and extensible neuroimaging data processing framework in Python. *Frontiers in Neuroinformatics*, *5*, 13. https://doi.org/10.3389/fninf.2011.00013

Gorgolewski, K. J., Durnez, J., & Poldrack, R. A. (2017). Preprocessed Consortium for Neuropsychiatric Phenomics dataset. *F100Research*, *6*, 1262. https://doi.org/10.12688/f1000research.11964.2

Gorgolewski, K. J., Esteban, O., Markiewicz, C. J., Ziegler, E., Ellis, D. G., Notter, M. P., Jarecka, D., et al. (2018). Nipype. *Software*. https://doi.org/10.5281/zenodo.596855

Greve, D. N., & Fischl, B. (2009). Accurate and robust brain image alignment using boundary-based registration. *NeuroImage, 48*(1), 63–72. https://doi.org/10.1016/j.neuroimage.2009.06.060

Jenkinson, M., Bannister, P., Brady, M., & Smith, S. (2002). Improved optimization for the robust and accurate linear registration and motion correction of brain images. *NeuroImage, 17*(2), 825–841. https://doi.org/10.1006/nimg.2002.1132

Jenkinson, M., & Smtih, S. (2001). A global optimisation method for robust affine registration of brain images. *Medical Image Analysis, 5*(2), 143-156. https://doi.org/10.1016/s1361-8415(01)00036-6

Lanczos, C. (1964). Evaluation of noisy data. *Journal of the Society for Industrial and Applied Mathematics Series B Numerical Analysis*, *1*(1), 76–85. https://doi.org/10.1137/0701007

Mumford, J. A., Poline, J.-B., & Poldrack, R. A. (2015). Orthogonalization of regressors in fMRI models. *PLoS ONE, 10*(4), e0126255. https://doi.org/10.1371/journal.pone.0126255

Patriat, R., Reynolds, R. C., & Birn, R. M. (2017). An improved model of motion-related signal changes in fMRI. *NeuroImage, 144*(Part A), 74–82. https://doi.org/10.1016/j.neuroimage.2016.08.051

Power, J. D., Mitra, A., Laumann, T. O., Snyder, A. Z., Schlaggar, B. L., & Petersen, S. E. (2014). Methods to detect, characterize, and remove motion artifact in resting state fMRI. *NeuroImage, 84*(Supplement C), 320–341. https://doi.org/10.1016/j.neuroimage.2013.08.048

Satterthwaite, T. D., Elliott, M. A., Gerraty, R. T., Ruparel, K., Loughead, J., Calkins, M. E., Eickhoff, S. B., Hakonarson, H., Gur, R. C., Gur, R. E., & Bassett, D. S. (2013). An improved framework for confound regression and filtering for control of motion artifact in the preprocessing of resting-state functional connectivity data. *NeuroImage, 64*(1), 240-256. https://doi.org/10.1016/j.neuroimage.2012.08.052

Souter, N. E., Bhagwat, N., Racey, C., Wilkinson, R., Duncan, N. W., Samuel, G., Lannelongue, L., Selvan, R., & Rae, C. (2024). Measuring and reducing the carbon footprint of fMRI preprocessing in fMRIPrep. *Human Brain Mapping*, *45*(12), e70003. https://doi.org/10.1002/hbm.70003

Tustison, N. J., Avants, B. B., Cook, P. A., Zheng, Y., Egan, A., Yushkevich, P. A., & Gee, J. C. (2010). N4ITK: improved N3 bias correction. *IEEE Transactions on Medical Imaging*, *29*(6), 1310-1320. https://doi.org/10.1109/TMI.2010.2046908

Zhang, Y., Brady, M., & Smith, S. (2001). Segmentation of brain MR images through a hidden Markov random field model and the expectation-maximization algorithm. *IEEE Transactions on Medical Imaging*, *20*(1), 45-57. https://doi.org/10.1109/42.906424
